# Exploring the link between work-related psychosocial factors and professional quality of life among ethiopian healthcare workers: Insights from structural equation modelling analyses

**DOI:** 10.1371/journal.pone.0319870

**Published:** 2025-03-26

**Authors:** Yitagesu Habtu, Abera Kumie, Medhine Selamu, Mirgissa Kaba, Hidenori Harada, Eshetu Girma

**Affiliations:** 1 Department of Preventive Medicine, School of Public Health, Addis Ababa University, Addis Ababa, Ethiopia; 2 Department of Mental Health Epidemiology, School of Nursing and Midwifery, Addis Ababa University, Addis Ababa, Ethiopia; 3 Graduate School of Asian and African Area Studies, Kyoto University, Kyoto, Japan; 4 African Population and Health Research Center, Nairobi Kenya; Alexandria University Faculty of Nursing, EGYPT

## Abstract

**Background:**

Work-related psychosocial factors increase the likelihood of poor professional quality of life (PQoL) outcomes, which are composed of three subscales burnout (BO), compassion fatigue(CF), and low compassion satisfaction (CS). However, studies on the impact of work-related psychosocial factors and the mediating role of workplace social support on PQoL in Ethiopian healthcare workers are limited. Therefore, our study aimed to explore the link between work-related factors and three subscales of PQoL, and assess the mediational role of workplace social support.

**Methods:**

We used a cross-sectional study design in selected public hospitals in Central and Southern Ethiopia between January and February 2023. We used a stratified random multistage sampling technique to select participants. We collected data on our endogenous variables using the PQoL-9 and data on exogenous work-related psychosocial variables using psychometrically validated scales. The data were entered using Epi-info 7 and exported to JAMOVI 2.4.14 for structural equation modelling.

**Results:**

A total of 1426 healthcare workers participated in the study. Among health workers, 25.5% experienced burnout above the third quartile, 24.8% had compassion fatigue above the third quartile, and nearly half scored below the third quartile for compassion satisfaction. Healthcare workers’ exposure to higher job demands (β=0.186) and work-family conflict (β =  0.306) were positively associated with BO, while decision latitude (β = -0.133), social support (β = -0.178), and job rewards (β = -0.170) were negatively associated. Decision latitude (β = -0.186), job rewards (β = -0.227), and social support (β = -0.152) are negatively associated, and work-family conflict (β =  0.367), and job effort (β =  0.067) positively associated with CF. Regarding CS, social support (β =  0.305), decision latitude (β = 0.262), and job rewards (β = 0.068) were positively associated, while work-family conflict (β =  -0.199) was negatively associated.

**Conclusion:**

Our study highlighted the importance of promoting workplace interventions among healthcare workers to reduce BO, and CF, and increase CS. Various job rewarding strategies including revising current salary evaluation and grading systems, incentive packages, and recognition systems are required to improve health workers’ PQoL. Interventions focusing on work-family balance, workload management skills, technical job decision latitude skills and task force allocation may be required to optimize job demands and controls.

## Background

Professional quality of life (PQoL) refers to the overall well-being of individuals in their professional roles, encompassing both positive and negative aspects of their work experiences [1, 2]. The concept of Professional Quality of Life (PQoL) was developed by Charles R. Figley, along with specific measures created in collaboration with Beth Hudnall Stamm to assess the positive and negative experiences of professionals in caregiving roles [1–4]. Healthcare workers help individuals who have been exposed to traumatic stressors, such as patient death and dying, patients suffering from serious pain or injury, grieving families, and patients facing critical financial or resource shortages, encountering essential decisions with limited information and resources [1–3]. They also deal with situations involving violence and danger, the aftermath of errors, complicated medical conditions, uncertain diagnoses, and unpredictable outcomes. Furthermore, healthcare workers may undergo a process of over-identifying with their patients and putting themselves in patients’ shoes, which can further complicate their emotional experiences.

Due to prolonged exposure to such direct and indirect traumatic events, healthcare workers may be at a greater risk of developing both negative and positive symptoms, depending on personal resources. Negative secondary outcomes consist of burnout (BO) syndrome and compassion fatigue (CF) [1, 2]. The term compassion fatigue is interchangeably used with secondary traumatic stress (STS) [1,2,4]. Because both describe emotional exhaustion and trauma-related symptoms arising from prolonged exposure to others’ suffering [1–3]. The BO subscale in PQoL [1, 2] focuses on work-related exhaustion, ineffectiveness and frustration by work demands. Conversely, the positive feelings related to helping and/or caring for patients and others are referred to as Compassion Satisfaction (CS) [1, 2]. The negative aspects of professional quality of life affect healthcare workers, their families, close others, the care they provide, and their organisation. Whereas, the positive aspects of helping reflect the selfless humanity of healthcare workers, who take pride in supporting patients. Policymakers and researchers can also contribute to enhancing these positive experiences by promoting practices that improve the well-being and productivity of healthcare workers [1, 2].

Despite a growing interest in this topic over the past 30 years [5], studies on complex psychosocial work-related factors that could be associated with both the negative and positive aspects of providing care remain under-researched [5, 6], particularly in the context of low-income countries. Even with an unequal distribution of traumatic work events, there is evidence of a disproportionate prevalence of burnout symptoms and secondary traumatic stress symptoms among health workers [5]. For example, a meta-analysis indicated that the overall prevalence rates of compassion fatigue, burnout, and compassion satisfaction among nurses are 52.55%, 51.98%, and 47.55%, respectively [5]. Similarly, another meta-analysis found that the overall prevalence rates of CS, BO, and CF among oncology nurses were 22.89%, 62.79%, and 66.84%, respectively[7]. Likewise, the overall prevalence of CF and BO was 65% among emergency nurses [6], and 54.60% among physicians [8], respectively. Moreover, a meta-analysis indicated that the overall prevalence rate of BO symptoms among all healthcare workers was 52% [9]. The most affected healthcare workers were from Asia in those meta-analysis studies [5,6]. Studies from Africa are rarely included in those meta-analyses, and almost all studies are targeted at a specific category of healthcare professions such as nursing or physicians. The overall prevalence of compassion fatigue and burnout was 65% among emergency nurses (6) and 54.60% among physicians (7), respectively.

In Ethiopia, while other key dimensions of PQOL remain unclear, BO is recognised as a significant mental well-being issue among healthcare workers, affecting a substantial proportion, ranging from 15.1% to 66.6% [10–15]. Nonetheless, burnout is not the only measure of emotional health in the working population, including health workers. Studies on other dimensions of PQoL, such as compassion satisfaction and compassion fatigue, have rarely been conducted among health workers. However, recent studies among nurses found a low mean score for compassion satisfaction [16] and a higher proportion (63.4%) of nurses with low compassion dissatisfaction [14]. Similarly, one study reported that 65.4% of health workers were affected by secondary traumatic stress [14], and another reported a higher mean score of secondary traumatic stress [16] (which may imply a higher score of compassion fatigue) among nurses.

The Ethiopian government recognises the importance of Compassionate, Respectful, and Caring (CRC) practices [17] among healthcare workers to enhance healthcare quality and patient-centered care. The health sector transformation plan also prioritizes the cultivation of motivated, competent, and compassionate (MCC) workers. While focusing on developing MCC is valuable for improving patient-centred care and healthcare quality, it is equally important to address the professional quality of life (PQoL) of health workers, as this is crucial for the overall well-being of the health workforce. Studies have indicated that high levels of compassion fatigue and low compassion satisfaction can reduce clinical competency [18], whereas burnout increases the risk of compassion fatigue [19]. Hence, it is vital to enhance the psychological and mental well-being of those caring for others by fostering a supportive work environment. To this end, further evidence-based organisational prevention programs are required to maximise health workers’ well-being by promoting compassion satisfaction (CS) and reducing the risk of developing compassion fatigue and burnout. To do so, gathering evidence on professional quality of life and its work-related psychosocial factors will provide information for improving the well-being of the healthcare workforce.

Professional quality of life is a multidimensional and complex phenomenon influenced by work-related factors, individual health worker behaviour, and exposure to primary and secondary trauma. Both the earliest biopsychosocial model [20] and contemporary psychiatrist [21] emphasise the need for further exploration of complex biopsychosocial factors including work-related factors. Similar to factors of other mental health outcomes, investigating work-related psychosocial factors of PQoL would provide information for targeted intervention. To investigate these work-related psychosocial factors of well-being, various theories in occupational settings have been used to explain how exposure to adverse working conditions affects the mental well-being of working populations including health workers. Since burnout, compassion fatigue, and compassion satisfaction are key mental health outcomes of PQoL, their link with work-related psychosocial factors can be explained by previous work-related stress models. Of those models, the job-demand control or decision latitude (JDC) model developed by Rober Karasek [22] and later expanded by Töres Theorell to include social support (JDC-S)[23], effort-reward imbalance (ERI)[24], job demand resources theory (JDR) [25], and work-family conflict or imbalance theory [26] are the most commonly used frameworks for explaining the link between work conditions and mental health outcomes, including the key constructs of PQOL(burnout, compassion fatigue, and compassion satisfaction). Various empirical studies have explored the link between work-related psychosocial factors using these theoretical models; however, almost all studies have been conducted in high and middle-income countries.

The association between work-related psychosocial stressors and constructs of PQOL, particularly burnout, has long been documented in biomedical and sociological studies [25,27–30]. According to JDC-S [22] and JDR[25], empirical studies show that higher job demands, low control or decision latitude, and low social support increase the risk of burnout [27, 28]. Employees with higher scores on job demands, lower control or decision latitude, and lower social support were more likely to report maladaptive coping strategies such as self-undermining, which can lead to job dissatisfaction[25] and may harm compassion satisfaction. Higher scores on job demands positively predicted burnout dimensions of emotional exhaustion (EE) and depersonalization (DP) and negatively predicted personal accomplishment (PA) [28,31], and compassion [32]. Low job control increases the likelihood of EE, DP, and CF [32], and decreases the likelihood of PA [25,28,31]. Furthermore, low social support increases the likelihood of burnout dimensions such as EE, DP, and reduced PA [25,31,33–38]. Low social support also increases the likelihood of compassion fatigue, or secondary trauma forms [25,33,34,37,39,40], whereas higher social support increases the likelihood of compassion satisfaction[37]. However, most of the studies in our literature review measured burnout in a slightly different from the way burnout is defined in the PQoL framework. They used the Maslach Burnout Inventory (MBI)[41], which explicitly includes three depersonalization (EE), depersonalisation (DP), and reduced personal accomplishment (PA) or professional inefficacy.

The effort-reward imbalance (ERI) model is a widely studied framework in work-related stress, and health studies including mental health, primarily focusing on the interaction of two extrinsic factors, “Efforts” and “rewards” [24]. The model was later expanded to include an intrinsic factor termed “over-commitment”[42]. According to the ERI model [24], higher individual efforts such as energy, time, and commitment an individual invested in their work combined with low rewards (which encompass material gains such as salary and benefits as well as psychosocial rewards such as recognition, job security, and career advancement) leads the working population to experience various mental and physical health issues, dimensions of quality of life (PQoL). Empirical studies based on this theory or using parts of its constructs have indicated that a higher job effort score on the ERI increases the likelihood of burnout syndromes [31,43,44]. Similarly, a lower job reward score increases the likelihood of burnout, and compassion fatigue [40], while reducing compassion satisfaction [44]. A higher effort-reward ratio corresponds to an increased risk of burnout [45, 46], and a decrease in compassion satisfaction [32].

Work-family conflict (WFC) is another theory that explains how poor work and family interfaces can negatively impact mental health [26]. Similarly, higher work-family conflict is associated with the likelihood of experiencing burnout [36,43,47,48]. One study showed that the work-family conflict score increased the likelihood of experiencing EE and decreased PA [49] of the dimensions of burnout. Additionally, a work-family imbalance has been shown to decrease CS [50].

Social support specifically supported by co-workers and supervisors or managers could mediate the relationship between work-related psychosocial factors and mental health according to the JDC-S model [23]. The JDC-S model did not specifically theorize the relationship between work-related factors, and PQoL dimensions as defined in PQoL frameworks [1, 2] among health workers. Similar to mechanisms in other mental well-being issues, social support can help health workers appraise work stressors and provide instrumental, informational, and emotional support. This, in turn, buffers the effect of work-related factors on PQoL. Despite the differences in predictors and outcomes in the current study, social support plays a mediating role in the relationship between work-related factors and mental health outcomes [23]. Additionally, studies have indicated the mediating roles of social support in the relationship between various work-related factors and health outcomes, such as workplace violence and turnover intentions among nurses [51], workplace bullying and health [52], job stress and mental well-being [53], ERI and quality of life [54], stress overload, fatigue, and turnover intention [55]. These findings underscore the importance of assessing the mediating effect of social support, despite differences in measurement approaches and health outcomes in our study. Although studies specifically assessing the mediating effect of social support in the relationship between our hypothesised work-related psychosocial factors and PQoL are limited, our study aims to explore this mediation to better interpret our findings.

It is also crucial to recognise that the dimensions of PQoL are significantly interrelated, one element may affect another. For instance, compassion fatigue positively predicts burnout [38,56], and burnout negatively predicts compassion satisfaction [44,57,58]. Again, burnout positively predicts compassion fatigue [44,57,58]. For this reason, the scores of each PQoL construct should be co-variated to control for the effect of one dimension on another within the framework of PQoL. This approach helps assess the effect of work-related factors on each dimension of PQoL.

To date, studies testing complex associations between work-related psychosocial stressors and PQoL dimensions are scarce in low and middle-income countries in general, and Ethiopia in particular. This presents an opportunity to provide evidence for designing workplace mental health strategies and policies aimed at improving well-being, productivity, and quality of healthcare services. Therefore, our study aimed to assess the association between work-related psychosocial factors and PQoL, as well as the mediating effect of social support in the relationship between work-related psychosocial factors and PQoL using structural equation modelling.

**Fig 1:**
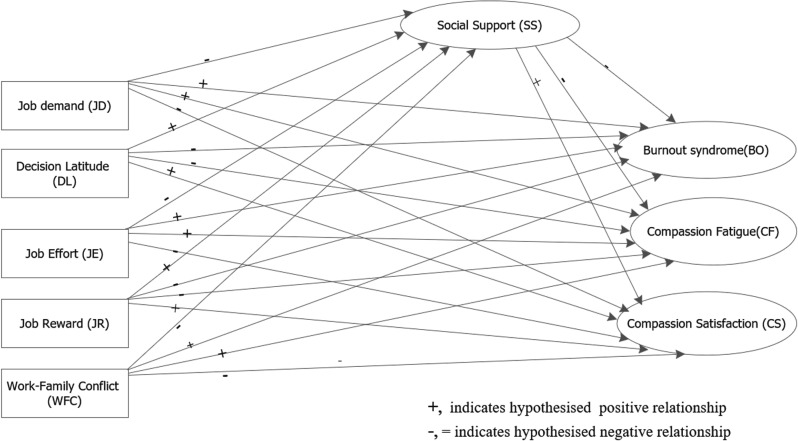
Hypothesized model of the relationship between work-related psychosocial factors and PQoL among healthcare workers, February 2023.

We formulated the following hypotheses based on athorough review of previously proposed theories and empirical studies: (1) higher job demands score increase the likelihood of burnout, compassion fatigue, and low compassion satisfaction;(2) lower job control or decision latitude score significantly increases burnout, compassion fatigue, and decreases compassion satisfaction;(3) lower social support score significantly increases burnout, compassion fatigue, and decreases compassion satisfaction; (4) higher extrinsic job effort score significantly increases burnout, compassion fatigue, and decreases compassion satisfaction; (5) lower job reward score significantly increases burnout, compassion fatigue, and decreases compassion satisfaction; (6) higher work-family conflict score increases burnout, compassion fatigue, and decreases compassion satisfaction; and (7) social support score mediates the relationships between job demands, job control, job effort, job reward, work-family conflict, and professional quality of life (PQoL). Fig 1 displays our conceptual model from previous studies to test our hypotheses in the context of Ethiopian health workers.

## Methods

### Study design, settings and period

We conducted a cross-sectional study between January 16 and February 28, 2023, in nine hospitals located across six zones in the Central and Southern Regions of Ethiopia. The administrative zones include Hadiya, Halaba, Kembata, Gurage, Wolaita, and Silitie. Hospitals serve multi-ethnic, multi-cultural, and multi-lingual populations. The administrative zones are located 132 to 328 kilometres from Addis Ababa, with a total population of approximately more than 9,201,127.

Ethiopia’s health system is organized into three tiers [17]: primary, secondary, and tertiary care. Primary care includes health posts, centres, and hospitals, with services ranging from preventive care at the community level to inpatient and surgical care. Secondary care is provided by general hospitals that receive referrals from primary facilities, while tertiary care involves specialized hospitals for advanced treatments and referrals from both general and some primary hospitals. During data collection, there were 317 functional public health facilities: 293 health centres, sixteen primary hospitals, four general hospitals, and four tertiary hospitals (one specialised and three referral hospitals), employing over 7,790 healthcare professionals, including 3,905 nurses, 1,467 midwives, 1,138 public health officers, 764 general practitioners, and 516 laboratory professionals.

The study targeted primary and higher-level hospitals, where psychosocial factors like higher job demands in various forms, job control, social support, effort-reward imbalance, and work-family conflict are potentially increasing the vulnerability of health workers to deteriorating professional quality of life (PQoL) among health workers. Data were collected from health workers working in nine selected hospitals: three tertiary hospitals, two general hospitals, and four primary hospitals.

### Source and study population

All health workers directly involved in clinical or patient care and paramedic services at those nine hospitals comprised the source population. Despite differences in the depth and scope of care, both healthcare workers who are directly involved in the clinical aspects and other paramedic activities could experience positive and negative aspects of their professional quality of life. Participants who were included from each randomly selected healthcare facility in each stratum composed the study population.

### Sample size determination

This study was part of a large PhD project with other study objectives having distinct research questions, including the current study, in the same target population. Therefore, we calculated separate minimum sample sizes using Westland JC’s [59] lower-bound sample size formula for structural equation modelling (SEM).

First, we assumed ten latent constructs with a maximum of ten free parameters at the initial stage of the study, a priori sample size determination. Accordingly, we had ten latent variables corresponding to the number of observed variables in parentheses: job demand (5), decision latitude (6), social support (6), work-family conflict (6), job effort (effort (3), job reward (7), perceived occupational stress (4), occupational depression (9), job anxiety (15), and professional quality of life (9) and a maximum of 16 regression parameters to increase the sample size.

Therefore, we calculated sample size for 86 observed variables (70 observed, 16 observed parameters) and 10 latent variables, 80% statistical power, 0.3 effect size, and 0.05 significance level. This resulted in a minimum number of 928 health workers. Applying a 1.5 design effect for multistage sampling error and a 10% allowance for nonresponse based on participation rates in previous studies in Ethiopia among healthcare workers, the final minimum recommended sample size was 1,531 health workers.

Secondly, we dropped three latent variables mentioned above (perceived occupational stress, occupational depression, and job anxiety), which are not parts of the current study objective. And, we substituted them with subscales of PQoL at the initial stage of the proposal, we calculated our sample size for the current study using the same assumptions, design effect, and non-response rate. However, the sample size was much lower than the sample size calculated for another PhD study objective (1531 health workers). So, we used this large sample size to model our latent constructs: social support (6 items), work-family conflict (6 items), job effort (3 items), job reward (7 items), and professional quality of life (BO = 3 items, CF = 3 items, and CS = 3 items) to estimate parameters in the structural equation model.

### Variables of the study

In structural equation modelling language, we have two variables: endogenous and exogenous variables. All variables are based on the theoretical frameworks, and or previous empirical studies as depicted in our literature review (Fig 1). Our endogenous variables include professional quality of life(PQoL) [2], which consists of three subdimensions; BO (3 items), CF(3 items), and CS (3 items), each measured by a validated tool [60] used for this study. Social support (6 items) is also an endogenous variable, a mediator variable adapted from the job-demand control-social support (JDCS) [22] model. Our exogenous variables include work-related psychosocial factors such as job demands, and job control (or decision latitude) based on the job-demand control-social support (JDCS) [22] model. Additionally, job effort and job rewards according to the ERI model [24], and work-family conflict according to previous theory[26] are also exogenous variables.

### Measures

#### Participant demographic characteristics.

We collected demographic data including age, sex, education level, income, family size, ethnic group, marital status, professional category, and education using a standardized questionnaire. These participant characteristics have been previously described in a separate article focused on a different objective within the same PhD project, using the same sample population[61].

#### Professional quality of life (PQoL).

PQoL was measured using a 9-item PQoL Scale [60], which was developed based on versions IV and V of Stamn’s PQoL scale [2]. The scale consists of three subscales: BO (3 items), CS (3 items), and CF (3 items). Descriptive summaries of items of PQoL and all other measures are displayed in Table 1 of the result section.

**Table 1:** Descriptive statistics of measurement scales in health workers in central and southern Ethiopia, February 2023(n = 1426).

Constructs and items	Subscales	Mean	Std. Deviation	Skewness	Kurtosis
Professional Quality of Professional (PQoL)					
I think that I might have been affected by the traumatic stress of those I help.(CF1)	Compassion fatigue (CF)	1.72	1.166	1.477	.995
I feel trapped by my job as a helper. (BO1)	Burnout syndrome (BO)	2.14	1.317	.858	-.532
I like my work as a helper. (CS1)	Compassion satisfaction (CS)	3.64	1.223	-.660	-.525
I feel depressed because of the traumatic experiences of the people I help. (CF2)	CF	2.28	1.188	.579	-.480
My work makes me feel satisfied. (CS2)	CS	3.54	1.292	-.546	-.784
I feel worn out because of my work as a helper. (BO2)	BO	2.48	1.152	.262	-.695
I feel overwhelmed because the size of my workload seems endless.(BO3)	BO	2.11	1.100	.704	-.168
As a result of my helping, I have intrusive, frightening thoughts.(CF3)	CF	1.89	1.078	1.042	.333
I am happy that I chose to do this work.(CS3)	CS	3.30	1.342	-.292	-1.100
**Job Demand-Control- (Support) Scales**					
**Job demand(JD)**					
Does your job require you to work very fast? (JD1)	JD	2.92	0.80	-0.795	0.589
Does your job require you to work very hard? (JD2)	JD	2.73	0.88	-0.547	-0.305
Does your work demand too much effort? (JD3)	JD	2.73	0.91	-0.404	-0.578
Do you have enough time to do everything? (JD4)	JD	2.16	0.92	0.318	-0.791
Does your work often involve conflicting demands? (JD5)	JD	2.46	0.93	-0.272	-0.923
**Decision latitude or Job control (DL)**					
Do you have the possibility of learning new things through your work? (DL1)	DL	2.25	0.97	0.427	-0.776
Does your work demand a high level of skill or expertise? (DL2)	DL	2.81	0.95	-0.165	-1.050
Does your work require ingenuity? (DL3)	DL	2.31	0.96	0.339	-0.817
Do you have to do the same thing over and over again? (DL4)	DL	2.72	1.04	-0.118	-1.233
Do you have a choice in deciding how you do your work? (DL5)	DL	2.34	1.06	0.268	-1.144
Do you have a choice in deciding what you do at work? (DL6)	DL	2.37	1.07	0.228	-1.186
**Social Support (SS)**					
There is a calm and pleasant atmosphere where I work. (SS1)	SS	2.68	0.87	-0.133	-0.671
There is a good spirit of unity. (SS2)	SS	3.14	0.72	-0.583	0.248
My colleagues are there for me.(SS3)	SS	3.10	0.74	-0.622	0.369
People understand that I can have a bad day. (SS4)	SS	3.12	0.72	-0.477	0.002
I get on well with my superiors. (SS5)	SS	3.03	0.78	-0.483	-0.184
I get on well with my colleagues. (SS6)	SS	3.14	0.74	-0.619	0.241
**Work-Family Conflict (WFC)**					
I have to miss family activities due to the amount of time I must spend on work responsibilities.(WFCt1)	Time-based	3.10	1.21	-0.155	-0.751
I am often so emotionally drained when I get home from work that it prevents me from contributing to my family. (WFCt2)	Time-based	2.48	1.21	0.340	-0.823
The behaviours I perform that make me effective at work do not help me to be a better parent and spouse. (WFCs1)	Strain-based	3.00	1.23	-0.125	-0.871
I have to miss work activities due to the amount of time I must spend on family responsibilities. (WFCs2)	Strain -based	2.29	1.22	0.560	-0.67
Because I am often stressed by family responsibilities, I have a hard time concentrating on my work. (WFCb1)	Behaviour-based	2.59	1.22	0.249	-0.872
Behaviour that is effective and necessary for me at home would be counterproductive at work. (WFCb2)	Behaviour-based	2.17	1.25	0.705	-0.621
Effort Reward Questionnaire					
**Job Effort(JE)**					
I have constant time pressure due to a heavy workload. (JE1)	Job effort	3.04	0.731	-0.3127	-0.384
I have many interruptions and disturbances while performing my job. (JE2)	Job effort	2.72	0.758	-0.0816	-0.402
Over the past few years, my job has become more and more demanding. (JE3)	Job effort	2.97	0.766	-0.3549	-0.297
**Job Reward(JR)**					
I receive the respect I deserve from my superior or a respected relevant person. (JR4)	Job esteem,	2.58	0.726	-0.2154	-0.201
My job promotion prospects are poor. (JR5)	job promotion	2.44	0.882	0.1097	-0.695
I have experienced or expect to experience undesirable changes in my work situation. (JR6)	job security,	2.5	0.742	-0.1153	-0.309
My job security is poor. (JR7)	job security	2.51	0.834	-0.1787	-0.559
Considering all my efforts and achievements, I receive the respect and prestige I deserve at work. (JR8)	Job esteem	2.49	0.781	-0.1039	-0.418
Considering all my efforts and achievements, my job promotion prospects are adequate. (JR9)	job promotion,	2.46	0.878	-0.0437	-0.716
Considering all my efforts and achievements, my salary/income is adequate (JR10)	job promotion, reward	2.37	0.852	-0.1418	-0.761

**Note 1:** WFCt, work-family conflict time based, WFCb, work-family conflict behaviour based; and WFCs, work-family conflict strain-based

**Note 2:** All effort items were reversed before the fitting both measurement model, and structural models, and items 5, item 6, and item 7 of the reward subscale were reversed before fitting both the measurement, and structural model

**Note 3:** All job demand and decision latitude items except JD4, and DL4 are reverse coded.

We used three items for measuring BO syndromes associated with feelings of depletion and difficulties in dealing with work (item: BO1), exhaustion (item: BO2) and a high workload (item: BO3). We measured CF using three items that assess the traumatic experiences of others (items: CF1 and CF2) and the symptoms that resemble those in traumatized individuals, such as intrusive, frightening thoughts (item: CF3). These items specifically target a theoretical approach to evaluating the impact of others’ traumatic experiences. We measured CS using three items that measure overall job satisfaction (items: CS1 and CS2) and specifically the satisfaction derived from helping others (item: CS3).

Participants rated on a five-point-Likert scale ranging from 1 (Never, indicating the experience doesn’t happen at all) to 5 (Very often, indicating that the experience occurred) for all three subscales). The tool has shown adequate reliability in the previous study in the working population including health workers from multicultural contexts in Spain, Argentina and Brazil [60]. The Cronbach’s α for BO was 0.810 for the first study and 0.834 for the second study. For CF, Cronbach’s α was 0.763 for the first study and 0.821 for the second study. For CS, Cronbach’s α was 0.737 for the first study and 0.843 for the second study [60].

#### Job demand-control-social support.

We measured job demand, job control or decision latitude, and social support using a validated 17-item job content questionnaire (JCQ-17)[62] with three subscales, which was selected from the long version of Karasek’s job demand social support scale [63]. This tool has shown good psychometric properties as validated in a huge sample of employees [62,64,65].

The tool consists of three subscales: psychological job demands (JD-5 items), job control or decision latitude (DL-6 items), and social support (SS-6 items). Psychological job demands include how health workers work fast (JD1), work intensity (JD2), work effort (JD3), the sufficiency of time to complete tasks (JD4) and conflicting demands (JD5). The job decision latitude or control includes the experience of learning new things (DL1), skill level to do the job (DL2), being creative (DL3), variety of work (DL4), choices of how to do the work (DL5) and choices of what to do at work (DL6). Participants rated on a four-point scale ranging from 1 (often, indicating that participants experience almost always) to 4 (never, indicating that they have never experienced). All items of job demands, and job control are reversed except JD4 (Do you have enough time to do everything?) and DL4 (Do you have to do the same thing over and over again?). In other words, the four-point scale later is reversed, where a response of “1” would be never and “4” would be often. After reversing all items except JD4, and DL4, the range of scores for job demand is from 5 to 20; a higher core score indicates greater job demand and the range of job latitude is from 6 to 24; the higher the score is, the greater the ability to decide or control the job.

Both job demands and decision latitude subscales have acceptable internal consistency according to previous validations [62,65]. The composite Cronbach’s α for job demand was 0.73 and 0.78 in previous studies [62,65]. Whereas the composite Cronbach’s α for the job decision latitude subscale was 0.75 and 0.78, in previous studies [62,65]. The subgroup by sex study also shows a Cronbach’s α for job demands, for males and females was between 0.70 and 0.85[64] while for job decision latitude has a low-reliability score Cronbach’s α for males and females was between 0.56 and 0.64 in previous study[64].

The social support subscales consist of six items that evaluate the pleasantness of the work atmosphere, the sense of unity, support from colleagues, helpfulness of colleagues, and relationship with both superiors and co-workers. Participants rated on a four-point scale ranging from 1 (Strongly disagree, indicating an intensity of support is low) to 4 (Strongly agree, indicating the intensity of support was high). The item scores range from 6 (lowest support) to 24 (highest support). The composite Cronbach’s α for social support was 0.84 and 0.86 [62,65]. The subgroup by sex study also shows a Cronbach’s α for social support for males and females was between 0.70 and 0.85 [64].

#### Job effort and rewards.

We measured job effort and rewards using the short version of the ERI questionnaire, which consists of 10 items (ERI-10) [66]. The job effort section (ERI-1 to ERI-3) addresses three key aspects: workload, job interruptions, and disturbances, as well as the demanding nature of job security over time. The job reward section covers ERI-4 to ERI-10, measuring esteem (ERI-4 and ERI-8), job security (ERI-6 and ERI-7), and job promotion (ERI-5, ERI-9 and ERI-10).

The participants responded on a four-point scale ranging from 1 (strongly disagree, indicating that the participants did not experience the described conditions to 4 (strongly agree, indicating that they did). The total score for job effort ranges from 3 (lowest effort) to 12 (highest effort). After reversing items 5, 6 and 7, the total score for job reward ranges from 7 (lowest reward score) to 28 (highest reward score). The ERI has been validated for the working population, including healthcare workers in high-income settings. It demonstrates good internal consistency, with Cronbach’s alpha values of 0.74 for effort and 0.79 for reward) [66], despite not being validated for low- and middle-income contexts.

#### Work-family conflict.

We measured work-family conflict using the short version of the Work-Family Conflict Scale (WFC-6 items) [67]. This scale assesses the extent to which time demands, strain and behaviour-based conflicts related to work interfere with family responsibilities, interactions, and home life. Participants rated each item on a five-point scale, ranging from 1(often, indicating frequent experiences of work-family conflict issues) to 5 (never, indicating no such experience). After reverse-coding all items, the total scores ranged from 6 to 30. This construct demonstrated good reliability, with Cronbach’s α ranging from 0.83 to 87[68] in studies of employees, including healthcare workers.

### Adaptation and pretesting procedures

We adapted a self-report questionnaire for our setting to assess the work-related psychosocial and professional quality of life to ensure the items are relevant, accurate, and applicable to our healthcare work environments, and health workers understanding. We followed cross-cultural survey tool development steps for our measures along with previously validated measures to minimize biases during translation and administering into the local language, Amharic [69, 70].

Initially, two bilingual experts; one with a Master of Science degree in psychiatry and another with an MSc in environmental health; translated the previous English questionnaire validated for other contexts into Amharic. Next, these translators together with a corresponding author, reviewed and cross-checked each item against the original language for each item of specific constructs to reach a consensus. The finalised translation was then given to an English university lecturer, who holds a Master’s degree in Arts and is fluent in Amharic and knowledgeable in the study field, for back-translation into English. Another individual with a Master’s degree in Arts in English, fluent in Amharic, and familiar with the study area, conducted an additional back-translation.

Subsequently, the translated versions were reviewed by previous translators, one of the authors of the study, and three invited experts (one Master’s degree in Arts in psychology, one master’s of public health in epidemiology, and one Master’s degree in Arts in anthropology). This review was made to ensure local cultural, linguistic, experiential, and conceptual equivalence for each item of the constructs used. Finally, the revised questionnaire was administered to eighty healthcare workers from public health centres not included in the main study. They were instructed to provide feedback, which led to further refinements.

### Inclusion and exclusion criteria

Healthcare workers who had been engaged in clinical or paramedic activities currently working in each selected hospital, or who had transferred from other hospitals, were included in the study. Those who were on annual leave or who had changed professional activities and were assigned to other than clinical and paramedic activities for any reason during the data collection period were excluded. Additionally, healthcare workers who had been out of work for more than two weeks were not included in the study.

### Sampling procedures

We used a stratified random cluster multi-stage sampling method to select study participants. First, we stratified hospitals into three groups according to the current health tier system of the country: primary, general, and tertiary (teaching and referral) hospitals. Within each stratum, hospitals were considered as clusters, with the assumption that most units and healthcare activities within each group face similar work-related stressors. However, variability in the stratum of tertiary and referral hospitals would be assumed to have greater variability concerning work-related stressors due to diversified activities per speciality or professional category.

Second, to account for variability, we randomly selected 75% of tertiary hospitals, 50% of general hospitals, and 25% of primary hospitals. This process resulted in the random selection of three tertiary hospitals, two general hospitals and four primary hospitals from each category. The sample size was then proportionally distributed according to the size of each chosen hospital. Where applicable, samples designated for work units were further distributed across specialities.

Finally, healthcare workers from each unit of the selected hospitals were invited to participate until the allocated sample size was reached. Before data collection began, we gathered information about the number, specialities and roles of healthcare workers in each hospital unit. Unit managers or coordinators were informed about the study’s purpose before reaching out to each study participant. The entire multistage process was described in the previous study that involved a similar target population but different research questions and objectives within the larger PhD project [61].

### Data collection and quality assurance procedures

Data collection was conducted using a self-administered structured questionnaire. We trained eleven data collectors on the study’s objectives, measures, and sampling procedures, assisting participants with specific items or questions, distributing and returning questionnaires, and adhering to ethical considerations. After the training, each data collector pretested a minimum of seven questionnaires at healthcare centres near the public health facilities selected for the study. Feedback was gathered from the data collectors after one week. Based on this feedback, the questionnaire was finalized, duplicated, and distributed to each study facility.

Following the sampling procedures and exclusion criteria, data collectors contacted each health worker in every unit of the selected hospital during their working hours. Upon contact, participants were informed about the study’s purpose, benefits, risks, confidentiality, autonomy and justice. Written consent was obtained from each participating health worker on the first page of the questionnaire. Participants who were unable to complete the questionnaire due to a busy schedule were given additional time. Three facility unit heads or matrons were appointed for referral and above hospitals to assist the data collectors, while one was assigned to primary and general hospitals. This arrangement ensured that participants had adequate time to complete the questionnaire. If a healthcare worker misplaced their questionnaire and still wished to participate, the data collectors provided a replacement. Completed questionnaires were returned to the data collectors, who reviewed them for completeness and consistency.

### Data processing and analysis

Six individuals from health informatics entered the data using EPI-info version 7, then exported it to SPSS version 26, and merged it into a single dataset. The SPSS dataset was imported into JAMOVI version 2.4.14 for confirmatory factor analysis and structural equation modelling. Initially, we examined all latent variables to identify outliers and missing items. Responses from participants showing identical answers across all items within a construct were removed to address missing values for items. For continuous variables such as age, missing values were replaced with the mean, assuming that the data were missing completely at random.

We used descriptive statistics to describe our study population and examine the nature of the data distribution using statistics such as skewness. We described the level of professional quality of life by determining cut-off points for the subscales of professional quality of life (BO, CF STS, and CS) using percentiles. Accordingly, we categorize the scores as low, moderate, and high levels of each subscale for those who score below the 25^th^ percentile, the 25th to 75th percentile, and the 75th percentile.

We assessed reliability using ordinal Cronbach’s alpha values, as well as the discriminant validity of our latent variables using heterotrait-monotrait ratios of correlations between the subscales and scales. Before testing the structural model, we conducted a confirmatory factor analysis (CFA) to assess whether the factor loadings met acceptable thresholds and whether correlation coefficients were appropriate. We performed a separate confirmatory factor analysis based on previously validated tools and theoretical constructs. Before fitting measurement models and the structural models, all effort items were reversed. Additionally, the reward items (including items 5, 6, and 7) were also reversed. Similarly, all job demands items were reversed except for item 4. Additionally, item 4 of the decision latitude scale was also reversed before fitting both measurement and structural models. We removed factor loading with standardised coefficients below 0.5 and non-significant loading (p-value >  0.05). Additionally, items with correlations deemed theoretically or statistically implausible were excluded.

Because of violations of multivariate normality, and the ordinal nature of our data, we used the diagonal weighted least squares (DWLS) estimation method for both our measurement and structural models. Covarying was made between our exogenous variables (JD, DL, JE, JR, WFC) based on our previous theoretical knowledge or evidence-based relationship to control for the effect of one dimension on another. Additionally, subscales of our endogenous variable (BO, CF and CS) were co-variated to control for the effect of one dimension on another. Once the measurement model was completed, we proceeded to test the structural model following the same procedure. Model fitness indices are reported for both our separate CFA and the structural equation model, such as a Standardised Root Mean Square Residual (SRMR), Goodness of Fit Index (GFI), Adjusted Goodness of Fit Index (AGFI), Comparative Fit Index and Tucker‒Lewis Index (TLI). Both standardised and unstandardized estimations with a 95%CI and p-values were reported. Standardised beta coefficients as effect sizes along with 95%CI are reported and interpreted as how each work-related psychosocial factor affected each PQoL subscale.

### Ethics approval and consent to participate

The Institutional Review Board (IRB) of the College of Health Sciences of Addis Ababa University approved the study with a **protocol number: 080/22/SPH** under the main Ph. D work. The IRB operates by the Declaration of Helsinki, Good Clinical Practice(GCP) guidelines, the WHO Operating Guidelines for Ethical Review Committee, and the National Guideline for Research Ethics in Ethiopia. Study participants got clarification made by the data collectors, and reviewed information about the purpose, risks, benefits, confidentiality and autonomy of participation from the first page of the questionnaire. Following the information, study participants provide informed consent. Clarification was made not to use any identifiers such as names on the questionnaire, but only a code was recorded on the questionnaire. Data are saved in password-protected computers to be used only by the authors, when necessary.

## Results

### Study participants characteristics.

A total of 1426 health workers from various professions completed the self-administered questionnaire, yielding a 93.1% response rate. Participants had a median age of 30, with ages ranging from 22 to 58, and about one-sixth were aged 27 to 30. Approximately one-sixth of the participants were male. In terms of education and profession, 79.7% held a degree, and 48.5% were nurses. Participants were from three main ethnic groups: Hadiya (26.3%) followed by Wolaita(15.6%). Around half were Protestant, with 28.8% being Orthodox Christians. Most participants had a monthly income of 115 to 213 USD, with family sizes ranging from 1 to 4 or more individuals. Sociodemographic and behavioural variables, as well as individual and work environment characteristics, are briefly described in a previously published article [61]. That analysed the same sample population, but different research questions within the larger PhD project.

### Level of professional quality of life (PQoL)

The mean sum scores along with standard deviations (SD) for BO syndromes, CF and CS subscales were 6.73 (2.70), 5.88(2.58), and 10.49 (2.27), respectively. The distribution of health workers across quartiles for the burnout (BO) subscale was as follows: 23.8% fell into the lowest quartile (below the 25th percentile), 50.5% were in the interquartile range (25th to 75th percentile) and 25.7% were in the highest quartile (above the 75th percentile). For the compassion fatigue subscale, 25.7% of health workers were in the lowest quartile, 49.5% were in the interquartile range and 24.8% were in the highest quartile. Regarding the compassion satisfaction subscale, 19.7% were in the lowest quartile, 29.8% were in the interquartile range, and 50.4% were in the highest quartile.

### Measurement characteristics

Table 1 shows a descriptive summary of items for each construct: PQoL, JD, DL, SS, JE, JR, and WFC. The distribution of subscale items for each subscale or scale of endogenous and exogenous variables reveals varied patterns of skewness and kurtosis. Compassion Fatigue (CF) items exhibit moderate to high positive skewness, indicating that higher scores are less common, and a mix of leptokurtic and platykurtic distributions, suggesting a range of peak shapes. Burnout syndrome (BO) items are slightly to moderately positively skewed with predominantly platykurtic distributions, reflecting a generally flat shape with more frequent moderate scores. Compassion Satisfaction (CS) items are negatively skewed, indicating higher satisfaction is more common, and consistently platykurtic, pointing to a flat distribution shape. Job Demand (JD) items show slight negative skewness with both leptokurtic and platykurtic distributions, suggesting a mixed pattern of tail and peak shapes. Decision Latitude (DL) and Social Support (SS) items are mostly nearly symmetric or slightly skewed with platykurtic distributions, highlighting relatively flat and evenly spread responses. Work-family conflict (WFC) items vary from nearly symmetric to moderately positively skewed, all with platykurtic distributions, indicating flat shapes and a broad spread of responses. Job effort (JE) items exhibit slightly negatively skewed. In contrast, the job reward (JR) items generally exhibit nearly symmetrical and platykurtic distributions, reflecting a flat distribution shape and a balanced spread of scores.

### Separate testing work-related psychosocial and PQoL measures.

Before fitting the full structural model, we conducted CFA for each latent based on previous validation studies and theoretical frameworks. Specifically, we fitted the JD and DL subscales of the job demand-decision latitude model, the JE and JR subscales of the ERI model, and the BO, CF and CS subscales of PQoL together. Additionally, we separately fitted the social support subscale from the JDCS model and the WFC construct. Since our data violated assumptions of multivariate normality and linearity, and given the ordinal nature of all items of the scale items, we used the diagonally weighted least square (DWLS) estimation method with robust standard errors. The optimization was performed using the nonlinear minimisation with box constraints (NLMINB) method.

From the separate fitting of the measurement model, we found a non-significant low factor load (standardised factor load <  0.5 with p-value > 0.05) for two items (items 4 and 5) of the measured job demand subscales, and one item (item 1) of the measured decision latitude subscales. Accordingly, we removed those items from their corresponding subscales. Specifically, from the job demand subscale, item 4 (Do you have enough time to do everything?) and item 5 (Does your work often involve conflicting demands?) had factor loadings of 0.0467 and 0.470, respectively. From the job decision latitude subscale, item 1 (Do you have the possibility of learning new things through your work?) had a factor loading of 0.0808.

After removing problematic items, we found acceptable factor loadings for the remaining items of the JD and DL subscales. We also found adequate standardized factor loadings, reasonable correlation patterns, and acceptable model fit indices for the remaining items in each CFA model. The most common model fit indices, such as SRMR (ranging from 0.018 to 0.081), GFI (0.968 to 0.999), AGFI (0.913 to 0.997), CFI (0.963 to 0.995) and TLI (0.935 to 0.993), all indicated acceptable fit. However, the chi-square tests (χ²s) were significant for all models, which can indicate poor fit. This can be expected given the large sample size, and model complexity in which trivial differences between the observed and predicted covariances can lead to a significant chi-square. Additionally, with small sample sizes, the chi-square statistic may lack the power to detect model misfits. Furthermore, greater model complexity increases the likelihood of a significant χ² test, even when other fit indices suggest a good fit. Therefore, we reported multiple additional model fit indices, which indicated reasonable fit.

The correlations within the JD subscale ranged from 0.690 (JD1 and JD3) to 0.793 (JD1 and JD2), both within the acceptable threshold of 0.30. For the DL subscale, correlations varied from 0.248 (DL2 and DL6) to 0.801 (DL5 and DL6), all values were above 0.30. In the SS subscale, correlations ranged from 0.532 (SS1 and SS4) to 0.833 (SS5 and SS6). Within the JE subscale, correlations ranged from 0.497 (ERI2 and ERI3) to 0.571 (ERI1 and ERI2), while in the JR subscale, they ranged from 0.446 (ERI5 and ERI7) to 0.865 (ERI9 and ERI10). For WFC, item correlations varied between 0.517 (WFCt1 and WFCs1) and 0.841 (WFCt1 and WFCs1).

Correlations within items of the PQoL scale also were in the acceptable range despite weak correlation for some items in the subscales. Correlations within the BO subscale ranged from 0.300 (BO1 and BO2) to 0.694 (BO2 and BO3), and correlations within the CF ranged from 0.345 (CF2 and CF1) to 0.441 (CF1 and CF3). Correlations within the CS ranged from 0.572 (CS3 and CS2) to 0.762 (CS1 and CS2). As expected based on previous validations, negative correlations were observed between job demand and decision latitude items, job effort and reward items, compassion fatigue and compassion satisfaction, and burnout and compassion satisfaction. The observed and residual correlations and covariances between items of separate latent constructs are displayed in **supporting files** S1 Tables. Factor loadings and composite reliability indices, ordinal Cronbach’s α values for the latent constructs are within the acceptable ranges as displayed in **supporting file** S2 Tables.

#### Testing the full measurement model.

Once we completed a separate CFA for each latent construct, we fitted the full structural model by specifying the exogenous (JD, DL, JE, JR and WFC) and endogenous variables (SS as a mediator, and BO, CF, and CS of PQoL), as hypothesized in Fig 1. Although the overall chi-square test suggested poor model fit (χ²(666) = 5448, p <  0.001), other commonly used fit indices indicated an acceptable model fit: SRMR = 0.057, RMSEA = 0.071, GFI = 0.984, AGFI = 0.979, CFI =  0.983, and TLI = 0.981.

The composite reliability indices (Ordinal Cronbach’s α values) for the BO, CF, and CS of the PQoL subscales were 0.696, 0.718, and 0.849, respectively. Ordinal Cronbach’s α values for other constructs fall between 0.774 for the job reward scale and 0.939 for the social support scale. Except for the pair BO and CF, all other heterotrait-monotrait ratios of correlations range from 0.057 and 0.590, suggesting acceptable discriminant validity between the subscales and scales. However, the heterotrait-monotrait ratio of correlation between BO and CF is 1.091, exceeding the recommended threshold (typically below 0.85 or sometimes below 0.90). Despite potential overlapping between latent constructs, we also covariated these endogenous variables to minimise estimation errors. The average variance extracted (AVE) values for all scales are above the recommended threshold of 50%. It fell between 50.7% of the variance in CF items, and 74.5% of the variance in JD items, indicating a good convergent validity. According to the coefficient of determination R², approximately 52.1%, 49.2%, and 32.4% of the variance was explained by CF, BO, and CS, respectively. Only approximately 8.7% of the variance was explained by SS when mediating the relationship between work-related psychosocial factors (JD, DL, JE, JR and WFC). Despite the unavailability of cut-off points, a large proportion of variances was unexplained by each of the endogenous variables. Only approximately 8.7% of the variance was explained by SS when fittings as a mediator variable in the relationship between work-related psychosocial factors (JD, DL, JE, JR, and WFC) and PQoL subscales. Since no established cut-off points exist, a significant proportion of the variance remained unexplained by each endogenous variable.

The observed correlations between items of the same scale or subscale are within the acceptable ranges (above 0.300 between items of the same subscale or scale) with expected directional relationships as previous validation or their theoretical frameworks. The observed correlations between items within the same subscale or scale ranged as follows: CS (0.571 to 0.623), CF (0.347 to 0.587), BO (0.301 to 0.693), WFC (0.517 to 0.841), JE (0.561 to 0.825), JR (0.496 to 0.570) and SS (0.532 to 0.589). The observed and residual correlations and covariances are displayed in the **Supporting file** S3 Tables.

In the full CFA model, all items had adequate factor load (above 0.50). Table 2 presents the factor loadings, unstandardized and unstandardised estimates, standard errors (SEs), communalities, composite reliability indices and average variance extracted (AVE) values.

**Table 2:** Measurement model for the relationship between work-related psychosocial factors and PQoL among health workers in Central and Southern Ethiopia, 2023.

				95% CI		β 95% CI					Communality (R2)	Reliability, and validity statistics	
Latent	Observed	Estimate	SE	Lower	Upper	β	Lower	Upper	z	p		Ordinal Cronbach’s alpha	Average Variance Extracted (AVE)
JD	JD1	1.000	0.000	1.000	1.000	0.864	0.837	0.891			0.747	0.896	0.745
	JD2	1.065	0.025	1.015	1.114	0.920	0.898	0.942	42.10	< .001	0.846		
	JD3	0.927	0.021	0.886	0.968	0.801	0.774	0.828	44.30	< .001	0.641		
DL	DL2	1.000	0.000	1.000	1.000	0.713	0.676	0.751			0.509	0.796	0.564
	DL3	0.944	0.035	0.874	1.013	0.673	0.635	0.712	26.60	< .001	0.454		
	DL4	1.220	0.040	1.142	1.298	0.870	0.837	0.904	30.50	< .001	0.757		
	DL5	1.073	0.037	1.000	1.146	0.765	0.736	0.795	28.70	< .001	0.586		
	DL6	1.007	0.036	0.936	1.077	0.718	0.687	0.749	27.90	< .001	0.516		
JE	ERI1	1.000	0.000	1.000	1.000	0.910	0.845	0.975			0.828	0.774	0.546
	ERI2	0.725	0.052	0.623	0.827	0.660	0.602	0.718	13.90	< .001	0.435		
	ERI3	0.674	0.049	0.577	0.770	0.613	0.553	0.674	13.70	< .001	0.376		
JR	ERI4	1.000	0.000	1.000	1.000	0.639	0.604	0.674			0.408	0.925	0.664
	ERI5	1.166	0.037	1.094	1.238	0.745	0.718	0.772	31.60	< .001	0.555		
	ERI6	1.281	0.037	1.209	1.353	0.818	0.797	0.840	34.90	< .001	0.669		
	ERI7	1.198	0.038	1.122	1.273	0.765	0.739	0.791	31.20	< .001	0.585		
	ERI8	1.370	0.038	1.295	1.444	0.875	0.859	0.891	36.10	< .001	0.766		
	ERI9	1.478	0.042	1.395	1.561	0.944	0.932	0.956	35.00	< .001	0.892		
	ERI10	1.373	0.040	1.295	1.452	0.877	0.861	0.894	34.10	< .001	0.770		
WFC	WFCt1	1.000	0.000	1.000	1.000	0.845	0.826	0.865			0.714	0.923	0.719
	WFCt2	0.955	0.015	0.925	0.985	0.807	0.786	0.828	62.10	< .001	0.652		
	WFCs1	1.068	0.015	1.038	1.098	0.903	0.888	0.917	69.30	< .001	0.815		
	WFCs2	1.022	0.015	0.993	1.051	0.864	0.847	0.881	68.70	< .001	0.746		
	WFCb1	1.002	0.013	0.976	1.028	0.847	0.829	0.865	75.00	< .001	0.717		
	WFCb2	0.968	0.017	0.936	1.001	0.818	0.795	0.842	58.60	< .001	0.670		
BO	BO1	1.000	0.000	1.000	1.000	0.667	0.624	0.709			0.445	0.696	0.541
	BO2	1.092	0.043	1.008	1.175	0.728	0.698	0.758	25.50	< .001	0.530		
	BO3	1.208	0.042	1.126	1.290	0.806	0.780	0.831	28.90	< .001	0.649		
CF	CF1	1.000	0.000	1.000	1.000	0.759	0.715	0.802			0.575	0.718	0.507
	CF2	0.789	0.033	0.724	0.854	0.598	0.562	0.635	23.90	< .001	0.358		
	CF3	1.011	0.035	0.942	1.081	0.767	0.734	0.800	28.60	< .001	0.589		
CS	CS1	1.000	0.000	1.000	1.000	0.842	0.811	0.873			0.709	0.849	0.679
	CS2	0.986	0.031	0.926	1.047	0.831	0.801	0.860	32.10	< .001	0.690		
	CS3	0.950	0.029	0.893	1.007	0.800	0.766	0.834	32.60	< .001	0.640		
SS	SS1	1.000	0.000	1.000	1.000	0.617	0.584	0.651			0.381	0.939	0.735
	SS2	1.430	0.038	1.355	1.505	0.883	0.869	0.896	37.20	< .001	0.779		
	SS3	1.488	0.042	1.405	1.570	0.918	0.906	0.931	35.40	< .001	0.843		
	SS4	1.445	0.040	1.368	1.522	0.892	0.879	0.905	36.60	< .001	0.795		
	SS5	1.422	0.040	1.344	1.500	0.878	0.863	0.892	35.60	< .001	0.770		
	SS6	1.485	0.041	1.405	1.565	0.917	0.907	0.926	36.40	< .001	0.840		

Note: JE; Job Effort, JR; Job Reward, SS; Social support, Work-Family Conflict, JD, Job demand, DL; Decision Latitude; BO, Burnout syndrome; CF, Compassion fatigue, CS, Compassion Satisfaction, WFCt, work-family conflict time based, WFCb, work-family conflict behaviour based; and WFCs, work-family conflict strain base.

#### Structural link between work-related psychosocial factors and PQoL.

According to the hypothesized model in Fig 1, we used a standardised estimate with 95% to interpret the relationship between work-related psychosocial factors (JD, DL, JE, JR and WFC) and the three PQoL subscales (BO, CF, and CS of PQoL). Job demands (β=0.186, 95% CI: 0.124 to 0.249) and work-family conflict (β=0.306, 95% CI: 0.250 to 0.361) were positively associated with higher scores of burnout syndrome (BO). Conversely, decision latitude or job control (β = -0.133, 95% CI:-0.192 to -0.075), social support (β=-0.178, 95% CI:-0.228 to -0.128), and job rewards (β=-0.170, 95% CI: -0.229 to -0.111) were negatively associated with BO. Job effort (β=0.055, 95% CI:-0.003 to 0.113) did not show a significant impact on BO.

For compassion fatigue (CF), decision latitude (β=-0.186, 95% CI: -0.247 to -0.126) and job rewards (β = -0.227, 95% CI:-0.288 to -0.167) showed statistically significant negative associations. Social support (β = -0.152, 95% CI:-0.206 to -0.098) was also negatively associated with CF. In contrast, work-family conflict (β=0.367, 95% CI: 0.308 to 0.426) positively predicted CF, and job demands (β=0.027, 95% CI:-0.041 to 0.095) did not predict CF. Job effort (β =  0.067, 95% CI: 0.004 to 0.129) positively predicted CF.

In terms of compassion satisfaction (CS), social support (β=0.305, 95% CI: 0.254 to 0.355) Decision latitude (β =  0.262, 95% CI: 0.203 to 0.320), and job reward (β =  0.068, 95% CI: 0.005 to 0.131) were positively associated with higher CS. Conversely, work-family conflict (β=-0.199, 95% CI: -0.260 to -0.139) showed a negative association with CS. Job effort (β=-0.049, 95% CI: -0.113 to 0.014) was not significantly related to CS. The standardized coefficients and unstandardized coefficients with 95% CIs are displayed in Table 3.

**Table 3:** Parameters estimates for the relationship between work-related psychosocial factors and PQoL among health workers in Central Ethiopia and Southern Ethiopia Region, February 2023.

				95% Confidence Intervals			β 95% Confidence Intervals			
Dependent	Predictor	Estimate	SE	Lower	Upper	β	Lower	Upper	z	p
BO	JD	0.144	0.025	0.095	0.193	0.186	0.124	0.249	5.74	< .001
BO	DL	-0.125	0.029	-0.181	-0.068	-0.133	-0.192	-0.075	-4.34	< .001
BO	SS	-0.192	0.029	-0.249	-0.136	-0.178	-0.228	-0.128	-6.66	< .001
BO	JE	0.041	0.022	-0.002	0.084	0.055	-0.003	0.113	1.85	0.064
BO	JR	-0.178	0.032	-0.241	-0.115	-0.170	-0.229	-0.111	-5.52	< .001
BO	WFC	0.241	0.023	0.196	0.286	0.306	0.250	0.361	10.53	< .001
CF	JD	0.024	0.031	-0.036	0.084	0.027	-0.041	0.095	0.78	0.435
CF	DL	-0.198	0.034	-0.264	-0.132	-0.186	-0.247	-0.126	-5.88	< .001
CF	JE	0.056	0.027	0.003	0.108	0.067	0.004	0.129	2.08	0.038
CF	JR	-0.270	0.038	-0.345	-0.195	-0.227	-0.288	-0.167	-7.08	< .001
CF	WFC	0.330	0.028	0.274	0.385	0.367	0.308	0.426	11.73	< .001
CF	SS	-0.187	0.034	-0.254	-0.120	-0.152	-0.206	-0.098	-5.48	< .001
CS	JD	0.068	0.033	0.004	0.132	0.070	0.005	0.135	2.10	0.036
CS	DL	0.309	0.037	0.236	0.381	0.262	0.203	0.320	8.34	< .001
CS	JE	-0.046	0.030	-0.105	0.014	-0.049	-0.113	0.014	-1.51	0.132
CS	JR	0.090	0.042	0.007	0.173	0.068	0.005	0.131	2.12	0.034
CS	WFC	-0.199	0.031	-0.259	-0.138	-0.199	-0.260	-0.139	-6.43	< .001
CS	SS	0.416	0.038	0.341	0.490	0.305	0.254	0.355	10.96	< .001
SS	JD	-0.058	0.026	-0.109	-0.006	-0.081	-0.153	-0.009	-2.19	0.028
SS	DL	0.011	0.029	-0.045	0.067	0.013	-0.052	0.077	0.39	0.700
SS	JE	0.008	0.024	-0.039	0.054	0.011	-0.057	0.079	0.32	0.749
SS	JR	0.104	0.033	0.039	0.169	0.107	0.040	0.174	3.12	0.002
SS	WFC	-0.128	0.025	-0.178	-0.079	-0.176	-0.242	-0.109	-5.07	< .001

As shown in Table 4, we also assessed the indirect effect of each work-related factor on BO, CF, and CS through social support. Accordingly, the mediation analysis demonstrated that social support significantly mediated the relationships between our work-related psychosocial factors and professional quality of life (PQoL) subscales. Specifically, social support mediated the effect of job demands on burnout (β =  0.014, 95% CI: 0.001 to 0.028), compassion fatigue (β =  0.012, 95% CI: 0.000 to 0.024) and compassion satisfaction (β =  -0.025, 95% CI: -0.047 to -0.002). Social support also significantly mediated the effects of job rewards on burnout (β =  -0.019, 95% CI: -0.032 to -0.006), compassion fatigue (β =  -0.016, 95% CI: -0.028 to-0.004), and compassion satisfaction (β =  0.033, 95% CI: 0.011 to 0.054). Moreover, social support significantly mediated the effect of work-family conflict on burnout (β =  0.031, 95% CI: 0.017 to 0.046), compassion fatigue (β =  0.027, 95% CI: 0.013 to 0.040), and compassion satisfaction (β = -0.054, 95% CI: -0.076 to -0.032). However, social support did not mediate the effect of both DL and JE on all three subscales of PQoL(BO, CF and CS). The indirect effect of work-related factors on PQoL is displayed in Table 4.

**Table 4:** An indirect effect of work-related psychosocial factors on PQoL (BO, CF, and CS) among health workers in Central Ethiopia and Southern Ethiopia Region, 2023.

					95% Confidence Intervals			β 95% Confidence Intervals			
**Label**	**Description**	**Parameter**	**Estimate**	**SE**	**Lower**	**Upper**	**β**	**Lower**	**Upper**	**z**	**p**
IE1	JD → SS → BO	p58 * p42	0.011	0.005	0.001	0.022	0.014	0.001	0.028	2.08	0.038
IE2	JD → SS → CF	p58 * p51	0.011	0.005	0.000	0.021	0.012	0.000	0.024	1.98	0.048
IE3	JD → SS → CS	p58 * p57	-0.024	0.011	-0.046	-0.002	-0.025	-0.047	-0.002	-2.14	0.032
IE4	DL → SS → BO	p59 * p42	-0.002	0.005	-0.013	0.009	-0.002	-0.014	0.009	-0.39	0.700
IE5	DL → SS → CF	p59 * p51	-0.002	0.005	-0.012	0.008	-0.002	-0.012	0.008	-0.39	0.699
IE6	DL → SS → CS	p59 * p57	0.005	0.012	-0.019	0.028	0.004	-0.016	0.024	0.39	0.700
IE7	JE → SS → BO	p60 * p42	-0.001	0.005	-0.010	0.007	-0.002	-0.014	0.010	-0.32	0.750
IE8	JE → SS → CF	p60 * p51	-0.001	0.004	-0.010	0.007	-0.002	-0.012	0.009	-0.32	0.750
IE9	JE → SS → CS	p60 * p57	0.003	0.010	-0.016	0.022	0.003	-0.017	0.024	0.32	0.749
IE10	JR → SS → BO	p61 * p42	-0.020	0.007	-0.034	-0.006	-0.019	-0.032	-0.006	-2.76	0.006
IE11	JR → SS → CF	p61 * p51	-0.019	0.007	-0.034	-0.005	-0.016	-0.028	-0.004	-2.66	0.008
IE12	JR → SS → CS	p61 * p57	0.043	0.014	0.015	0.071	0.033	0.011	0.054	3.00	0.003
IE13	WFC → SS → BO	p62 * p42	0.025	0.006	0.013	0.036	0.031	0.017	0.046	4.14	< .001
IE14	WFC → SS → CF	p62 * p51	0.024	0.006	0.012	0.036	0.027	0.013	0.040	3.88	< .001
IE15	WFC → SS → CS	p62 * p57	-0.053	0.011	-0.075	-0.031	-0.054	-0.076	-0.032	-4.76	< .001

**Note 1:** IE1, Indirect Effect 1; BO, Burnout Syndrome; CF, Compassion Fatigue; CS, Compassion Satisfaction; JD, Job demand; JE; Job Effort; JR, Job Reward, DL, Decision Latitude; SS, Social support; and WFC, Work-family conflict,

**Note 2:** In notations like P58 and P42: P^58^ is the path coefficient from an exogenous variable (e.g., JD) to a mediator (SS); P^42^, represents the path coefficient from the mediator (SS) to an endogenous variable (BO); P^58 * ^P^42^ represents the combined indirect effect of JD on BO via SS; and the double arrows (e.g., “JD → SS → BO”) represents an indirect effect pathway where JD influence BO via SS as a mediator.

**Note 3**: Like in any regression concept, an estimate is an unstandardised coefficient representing the raw strength of the effect between two variables in their original units, while β is a standardized coefficient in our SEM analysis.

**Fig 2:**
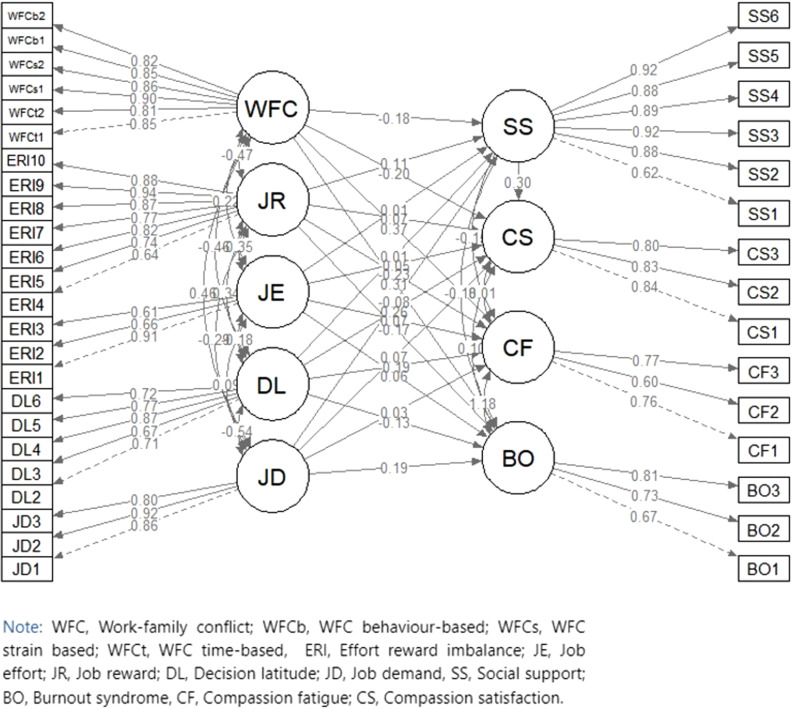
Path diagram of parameter estimates for the effect of work-related psychosocial factors on PQoL among health workers, 2023.

The path diagram for the overall structural equation modelling with standardized beta coefficients showing the association between exogenous and endogenous variables and their standardized factor loadings is displayed in Fig 2.

## Discussion

Our SEM analyses reveal significant relationships between work-related psychosocial factors and the three subscales of professional quality of life (PQoL) among healthcare workers. The results supported our hypotheses:(1) higher job demands and work-family conflict were linked to increased burnout levels;(2) lower decision latitude, social support, and job rewards were associated with higher burnout syndrome; (3) higher work-family conflict, coupled with lower decision latitude, social support, and job rewards, increased the likelihood of compassion fatigue; (4) greater social support, decision latitude, and job rewards were positively associated with compassion satisfaction, while higher work-family conflict correlated with lower compassion satisfaction; and (5) social support significantly mediated the effects of job demands, job rewards, and work-family conflict on burnout, compassion fatigue, and compassion satisfaction. However, our findings contradicted some of our hypotheses: higher job demands did not increase compassion satisfaction, nor was job demand significantly associated with compassion fatigue. Additionally, job effort was not significantly related to burnout syndrome or compassion satisfaction. Social support did not mediate the effects of decision latitude or job rewards on any PQoL subscale.

Our findings indicate that exposure to higher job demands increases burnout, a key dimension of PQoL. This occurs when health workers face excessive job demands but lack organizational or personal resources to manage them. As a result, their mental and physical reserves become depleted, leading to chronic work-related exhaustion, personal inefficacy, and frustration—ultimately resulting in burnout. Since burnout is a chronic mental health outcome, our findings align with the earliest job demand-decision latitude theory [22]. Additionally, previous studies supported our findings that exposure to higher job demands increases the likelihood of developing emotional exhaustion (EE) [28,31,71–74] and depersonalization (DP) [28,31,72,73]. Conversely, a higher job demand score is more likely to reduce personal accomplishment (PA) dimensions of burnout [28,31,72], and reduce compassion satisfaction [32,71]. However, most of the studies in our literature review measured burnout slightly differently from the way it is defined in the PQoL framework. Specifically, they used the Maslach Burnout Inventory (MBI)[41], which categorises burnout into three dimensions: depersonalization (EE), depersonalisation (DP), and reduced personal accomplishment (PA) or professional inefficacy [41]. Despite these differences, our findings convey the need to implement evidence-based interventions that optimize the job demands of healthcare settings. Doing so can mitigate burnout, support healthcare workers’ mental well-being, and enhance the quality of healthcare services.

Job demand did not significantly affect the CF of PQoL. This finding is contrary to our hypothesis, and against the previous studies that indicated that higher job demands are associated with increased CF [32,75]. A variety of explanations could be provided for this discrepancy. One possible reason could be due to omitting two job demand items due to low factor loading may introduce omission bias in our study, and the cross-sectional nature of our study could also contribute to false negative results. Additionally, despite facing higher job demands, health workers could remain committed to alleviating patient suffering or they could take job demands as opportunities for growth, learning, and mastery may experience higher CS rather than CF. Furthermore, individual differences in sense of meaning and purpose work engagement, and individual differences in resilience and coping strategies could also be cited for the discrepancy. Surprisingly, job demand positively predicted compassion satisfaction, which is also against our hypothesis, and previous studies [32,75]. Particularly, while job resources like social support, decision latitude, work-family conflict, and job reward affect the BO of PQoL health workers, it is complex to consider a scenario where higher job demands positively predict higher compassion satisfaction. This could be true if healthcare workers had developed some kind of personal resilience, strong work passion, strong self-care or coping mechanism or else. Item omission bias (omitting two job demand items due to low factor loading) may cause negative findings in our study. Additionally, social support may also play a moderation role in changing the direction and effect of job demand on the CS of the PQoL. Further research is needed to explore individual differences among healthcare workers for the above cases.

Our findings suggest that healthcare workers with higher job control or decision latitude had a lower likelihood of reporting BO and CF in the PQoL subscale scores. These findings are plausible, as supported by the earliest work-related psychosocial theories such as JDC-S[22], JDR [25], and other studies [27,28,31,32,75]. However, it is difficult to find studies for comparison in Ethiopian healthcare workers’ contexts. Higher job control or decision latitude among healthcare workers can buffer the effects of stress, reducing BO and CF, while increasing compassion satisfaction. In other words, the findings imply that greater autonomy and control, more decision-making power, and access to resources contribute to higher job satisfaction leading to lower level of compassion fatigue, and burnout. Therefore, policymakers and implementers should focus on enhancing decision latitudes or job control within healthcare settings to improve their well-being and achieve better patient care.

As per our hypotheses, lower social support (from managers or supervisors and co-workers) consistently increased the risk of burnout, compassion fatigue, and decreased compassion satisfaction. Our findings align with previous studies indicating that lower social support is associated with EE, DP and reduced PA subscales of burnout in healthcare workers [25,31,33–38]. Similarly, these findings are also consistent with studies that found lower social support increases the likelihood of CF [25,33,34,37,39,40,76], while higher social support increases the likelihood of compassion satisfaction [37]. Not only did social support directly impact all elements of PQoL, but also mediated the relationship between job demands, job rewards, and work-family conflict, and each of PQoL dimensions (BO, CF, and CS). In this mediational process, high job demand, and WFC scores lead to reduced social support, which in turn contributes to increased burnout. Conversely, increased rewards enhanced social support, thereby reducing burnout. Our findings are in agreement with studies indicating the mediating roles of social support in the relationship between various work-related factors and mental health outcomes [77–79]. Specifically, social support mediates the relationship between job demand, decision latitude and burnout[77–79], workplace violence and burnout [80], job stress and mental wellbeing [53], burnout and general health [81], ERI and quality of life [54], workplace bullying and health [52], workplace violence and turnover intentions among nurses [51], and stress and turnover intention [55]. The health outcomes in these studies differ from ours, but their findings remain applicable to PQoL dimensions, as these are recognized mental health outcomes.

Additionally, our findings suggest that low job reward scores consistently increased the likelihood of burnout syndrome, and compassion fatigue, and decreased compassion satisfaction in the PQoL. This aligns with the earliest ERI model[24], which theorises that higher individual effort coupled with lower rewards leads to various mental and physical issues within the working population. By implication, PQoL dimensions (burnout, compassion fatigue and compassion satisfaction) are among mental health outcomes though we couldn’t find similar studies with similar target populations for comparison. Studies also indicate that a lower job reward score of ERI increases the likelihood of burnout syndrome [40,82,8340,^82^,^83^], compassion fatigue [40,76], and reduced compassion satisfaction [44]. However, in our study, the job effort subscales of the ERI model [24] was not significantly associated with BO and CS, but it was significantly associated with CF in our study. These findings contradict earlier studies that indicate a higher job effort score of ERI increases the likelihood of burnout syndromes [31,43,44,82,83], and a higher job effort reduces compassion satisfaction [32]. However, we could not find studies conducted within the context of Ethiopian healthcare workers to compare with our findings. Here, we would like to remind readers that we could find slightly different results if we could have fitted the ratio of the job effort to job reward according to the ERI model [24], but our SEM model didn’t accommodate this score.

Both the job demand dimension of the JDCS model [22], and the job effort dimension of the ERI model[24] share common extrinsic work-related factors such as workload, time pressure, and physical and mental exercise. However, our findings indicated that JD is positively associated with BO, while JE is not. Various reasons can be presented for this paradox. In our case, the items of “time pressure” and “conflicting demands” were removed from the model due to lower factor loading (less than 0.5) during our CFA, which may have led to an underestimation of effect size. Additionally, the measurement approaches for BO in our study differ from those in other studies where the majority of studies used a long version of BO, a Mashlash burnout scale [41], and a long version of PQoL[2]. Another possible explanation is that social support may play a moderating role in the relationship between JE and BO. Furthermore, it could be due to healthcare workers having developed kinds of personal resilience, strong work passion, effective self-care or coping strategies that protect them from experiencing BO. Another paradox was that JD is positively associated with CS, while JE was not significantly associated with CS. In this context, higher effort does not necessarily lead healthcare workers to have lower compassion satisfaction. Instead, the effort-reward ratio may provide better information than effort or reward alone although the SEM model didn’t provide us a better fit when including the ratio. Moreover, studies with stronger designs and utilising a combination of these two theoretical models should be conducted to further explain such paradoxical findings.

Work-family conflict (WFC) is another theory that explains how poor interfaces between work and family can negatively affect mental health [26]. Our study found that the WFC score positively predicted both BO and CF scores and negatively predicted CS. These findings align with other studies indicating that higher work-family conflict increases the likelihood of experiencing burnout [36,43,47,48]. Another study also indicates that work-family imbalance decreases compassion satisfaction, another key dimension of PQoL[50]. To our knowledge, studies that indicate the relationship between WFC and compassion fatigue are rare, making comparison difficult, even though we hypothesised a relationship assuming that CF is a form of secondary traumatic stress like BO. Similarly, studies indicate the association between WFC and PQoL dimensions in Ethiopian health workers for comparison are scarce. It is important to recognize that work and family are two sides of the same coin. Conflicts arise in these life interfaces when work demands interfere with family life (or vice versa). If such conflicts remain chronic and are not resolved promptly, they increase the risk to mental well-being [26]. The scenario for BO, CF, and decreased CS didn’t differ from other mental health issues in health workers and their families. Therefore, our findings imply the need to promote work-life balance through flexible work arrangements, enhance social support to buffer the negative effects of work-family conflict and address work-family conflict. This helps improve health workers’ professional quality of life through sustainable organizational policies and procedures in the Ethiopian context.

Addressing PQoL requires consideration of complex work-related psychosocial causal pathways including work-related stressors. This aligns with the original biopsychosocial health model [20] as the PQoL dimensions should be considered as other common mental health outcomes. Therefore, despite a few exceptions, our study highlighted a significant association between work-related psychosocial factors and subdimensions of PQoL, as hypothesised. This provides insights for designing targeted control and prevention policies and professional practices to address healthcare workers’ burnout and compassion fatigue in Ethiopia. Healthcare policies, and systems should prioritise enhancing social support (from supervisors, managers, and colleagues), decision latitude/job control, and job rewards to enhance compassion satisfaction and mitigate burnout among healthcare workers. Implementing flexible work arrangements and ensuring adequate staffing levels could reduce job demands and work-family conflict, promoting the professional quality of healthcare workers and enabling them to provide quality healthcare. The healthcare system should incorporate regular assessments of work conditions and offer training on stress management and resilience-building for healthcare workers. Regarding our negative findings, such as the relationships between higher job demands and compassion satisfaction, job demand and compassion fatigue, job effort and burnout syndrome, and job effort and compassion satisfaction, additional studies with strong designs may be required to explore more about those relationships.

### Strengths and limitations of the study

To our knowledge, our study is the first to explore the complex causal relationships between work-related psychosocial factors and the subscales of PQoL among healthcare workers using a higher-order analysis that controls for confounding effects. We also measured PQoL among categories of healthcare workers, a topic that has not yet been addressed in previous studies in low and middle-income countries. This could contribute to targeted interventions aimed at improving PQoL benefiting healthcare workers by enhancing their professional well-being and the quality of healthcare provision. In line with our main purpose, the short-validated tools for PQoL could be adapted and scaled along with existing PQoL measures to other occupational contexts to investigate and implement appropriate measures. Additionally, employing a larger sample size one that could not have been calculated using conventional methods along with an intensive cultural adaptation of measurement, and posthoc checks for validity, and reliability before performing the SEM analysis, could provide us with conclusive evidence.

However, the cross-sectional nature of our study may limit our conclusions regarding the causal relationships between work-related factors and PQoL dimensions. We encountered difficulties in finding studies on the relationship between work-related factors and PQoL to compare our results with those from Ethiopia and other low-income settings. Despite our efforts for intensive cultural adaptation of the measurement tools, all cause-specific measures used in this study were not validated for the Ethiopian context. This limitation may account for the exclusion of job demand (2 items) and decision latitude (1 item) from the structural model due to poor factor loading, which may have led to omitted variable bias. Furthermore, due to economic and time constraints, the use of short versions of PQoL questionnaires may not capture other important aspects of PQoL. Certain individual and job-related psychosocial factors may be more prevalent in tertiary hospitals than primary hospitals because of higher proportion of tertiary hospitals were taken. However, stratifying by work-related stressor complexity across the nine hospitals in our multistage sampling process is unlikely to significantly compromise generalizability. This is because essential elements, such as resources for managing work stress are assumed to be equivalent across hospitals due to the absence of strong workplace well-being programs in the country. Consequently, our findings should still apply to healthcare workers in primary, secondary, and tertiary care settings, although some limitations exist.

## Conclusion

Our data suggest the need to design workplace interventions that enhance workplace social support in emotional, material, and technical aspects to reduce the likelihood of burnout (BO), and compassion fatigue (CF), and to increase compassion satisfaction (CS) elements of PQoL. Various job rewarding strategies including revising the current salary job evaluation and grading systems, incentive packages, and recognition systems are required to mitigate workers’ BO, CF, and low CS.

Additionally, task management and flexible work arrangements with medical material, and supply mobilization training, should be provided to healthcare workers to reduce job demands. Technical skill-based training could also enhance health workers’ abilities to manage their work effectively and exercise good decision-making latitude. Furthermore, training on work-family balance skills and other workplace well-being interventions may be necessary to alleviate work-family conflict, hereby reducing the BO, CF, and increasing CS of PQOL.

Further studies are needed to investigate the mediating and moderating role of social support in the relationship between work-related psychosocial factors and PQoL, and to explore additional explanations for negative findings (findings against our hypotheses), such as the relationships between higher job demands and compassion satisfaction, job demand and compassion fatigue, job effort and burnout syndrome, and job effort and compassion satisfaction.

## Supporting information

S1 Table Observed covariances and correlations (S1(a), S1(c), S1(e), S1(g), & S1(i)), and residual covariance, and correlation ((S1(b), S1(d), S1(f), S1(h), & S1(j)) between items of each latent measures in the study among healthcare workers in central and southern Ethiopia, 2023. (PDF)

S2 Table Separate measurement model, reliability, and validity indices (S2(a), and summary of additional information for CFA of the measurement model (S2(b)) in the study among health workers in central and southern Ethiopia, 2023. (PDF)

S3 Table Observed covariances and correlations (S3(a)), and residual covariance and correlations (S3(b)) between items of latent measures in the study among healthcare workers in central and southern Ethiopia, 2023. (PDF)
