# Supplementary material for: Exploring the link between work-related psychosocial factors and professional quality of life among ethiopian healthcare workers: Insights from structural equation modelling analyses
Source: PLoS One. 2025 Mar 26;20(3):e0319870. doi: 10.1371/journal.pone.0319870 (PMC11940713; doi:10.1371/journal.pone.0319870)
Supplement: S1 Table — (PDF) [file pone.0319870.s001.Pdf]

Table S1(a) to S1(j) . Observed covariances (Lower triangle) and correlations (upper triangle) between items of work-related psychosocial factors and PQoL latent measures among healthcare workers in central and southern Ethiopia, 2023.

S1(a) Table. Observed covariances (lower triangle) and correlations (upper triangle)

	BO1	BO2	BO3	CF1	CF2	CF3	CS1	CS2	CS3
BO1	1	0.298	0.304	0.678	0.272	0.298	-0.208	-0.216	-0.310
BO2	0.298	1	0.694	0.337	0.545	0.602	-0.111	-0.073	-0.228
BO3	0.304	0.694	1	0.365	0.558	0.747	-0.197	-0.161	-0.209
CF1	0.678	0.337	0.365	1	0.345	0.441	-0.314	-0.311	-0.369
CF2	0.272	0.545	0.558	0.345	1	0.588	-0.063	-0.062	-0.092
CF3	0.298	0.602	0.747	0.441	0.589	1	-0.256	-0.244	-0.239
CS1	-0.208	-0.111	-0.197	-0.314	-0.063	-0.256	1	0.762	0.626
CS2	-0.216	-0.073	-0.161	-0.311	-0.062	-0.244	0.762	1	0.572
CS3	-0.31	-0.228	-0.209	-0.369	-0.092	-0.239	0.626	0.572	1

S1 (b) Table. Residual covariances (lower triangle) and correlations (upper triangle)

	BO1	BO2	BO3	CF1	CF2	CF3	CS1	CS2	CS3
BO1	0	-0.133	-0.192	0.257	-0.116	-0.199	-0.052	-0.072	-0.185
BO2	-0.133	0	0.071	-0.193	0.058	-0.022	0.085	0.108	-0.072
BO3	-0.192	0.071	0	-0.244	-0.003	0.030	0.028	0.047	-0.029
CF1	0.257	-0.193	-0.244	0	-0.083	-0.107	-0.092	-0.106	-0.192
CF2	-0.116	0.058	-0.003	-0.083	0	0.084	0.141	0.127	0.071
CF3	-0.199	-0.022	0.030	-0.107	0.084	0	0.005	-0.003	-0.031
CS1	-0.052	0.085	0.028	-0.092	0.141	0.005	0	0.015	-0.017
CS2	-0.072	0.108	0.047	-0.106	0.127	-0.003	0.015	0	-0.023
CS3	-0.185	-0.072	-0.029	-0.192	0.071	-0.031	-0.017	-0.023	0

S1(c) Table. Observed covariances (lower triangle) and correlations (upper triangle)

	ERI1	ERI2	ERI3	ERI4	ERI5	ERI6	ERI7	ERI8	ERI9	ERI10
ERI1	1	0.571	0.536	-0.192	-0.322	-0.227	-0.237	-0.292	-0.306	-0.273
ERI2	0.571	1	0.497	-0.139	-0.215	-0.183	-0.231	-0.187	-0.182	-0.115
ERI3	0.536	0.497	1	-0.125	-0.209	-0.184	-0.188	-0.175	-0.197	-0.177
ERI4	-0.192	-0.139	-0.125	1	0.484	0.62	0.446	0.599	0.509	0.511
ERI5	-0.322	-0.215	-0.209	0.484	1	0.572	0.683	0.561	0.686	0.573
ERI6	-0.227	-0.183	-0.184	0.62	0.572	1	0.634	0.77	0.707	0.676
ERI7	-0.237	-0.231	-0.188	0.446	0.683	0.634	1	0.655	0.651	0.598
ERI8	-0.292	-0.187	-0.175	0.599	0.561	0.77	0.655	1	0.826	0.764
ERI9	-0.306	-0.182	-0.197	0.509	0.686	0.707	0.651	0.826	1	0.865
ERI10	-0.273	-0.115	-0.177	0.511	0.573	0.676	0.598	0.764	0. 865	1

S1(d) Table. Residual covariances (lower triangle) and correlations (upper triangle)

	ERI1	ERI2	ERI3	ERI4	ERI5	ERI6	ERI7	ERI8	ERI9	ERI10
ERI1	0	-0.011	-0.025	0.004	-0.099	0.024	-0.007	-0.024	-0.018	-0.007
ERI2	-0.011	0	0.051	0.017	-0.038	0.015	-0.049	0.025	0.045	0.096
ERI3	-0.025	0.051	0	0.025	-0.038	0.008	-0.012	0.030	0.023	0.028
ERI4	0.004	0.017	0.025	0	0.011	0.089	-0.041	0.031	-0.100	-0.055
ERI5	-0.099	-0.038	-0.038	0.011	0	-0.031	0.130	-0.083	-0.005	-0.069
ERI6	0.024	0.015	0.008	0.089	-0.031	0	0.012	0.045	-0.071	-0.046
ERI7	-0.007	-0.049	-0.012	-0.041	0.130	0.012	0	-0.010	-0.062	-0.064
ERI8	-0.024	0.025	0.030	0.031	-0.083	0.045	-0.010	0	-0.006	-0.008
ERI9	-0.018	0.045	0.023	-0.100	-0.005	-0.071	-0.062	-0.006	0	0.037
ERI10	-0.007	0.096	0.028	-0.055	-0.069	-0.046	-0.064	-0.008	0.037	0

S1(e) Table. Observed covariances (lower triangle) and correlations (upper triangle)

	JD1	JD2	JD3	DL2	DL3	DL4	DL5	DL6
JD1	1	0.793	0.690	-0.351	-0.455	-0.458	-0.279	-0.228
JD2	0.793	1	0.738	-0.398	-0.432	-0.456	-0.261	-0.174
JD3	0.690	0.738	1	-0.407	-0.377	-0.423	-0.198	-0.141
DL2	-0.351	-0.398	-0.407	1	0.450	0.556	0.292	0.248
DL3	-0.455	-0.432	-0.377	0.450	1	0.508	0.464	0.414
DL4	-0.458	-0.456	-0.423	0.556	0.508	1	0.335	0.301
DL5	-0.279	-0.261	-0.198	0.292	0.464	0.335	1	0.801
DL6	-0.228	-0.174	-0.141	0.248	0.414	0.301	0.801	1

S1(f). Table. Residual covariances (lower triangle) and correlations (upper triangle)

	JD1	JD2	JD3	DL2	DL3	DL4	DL5	DL6
JD1	0	-0.002	-0.008	-0.062	-0.132	-0.131	0.130	0.157
JD2	-0.002	0	0.007	-0.095	-0.095	-0.114	0.166	0.228
JD3	-0.008	0.007	0	-0.141	-0.081	-0.123	0.178	0.213
DL2	-0.062	-0.095	-0.141	0	0.042	0.142	-0.224	-0.238
DL3	-0.132	-0.095	-0.081	0.042	0	0.048	-0.110	-0.127
DL4	-0.131	-0.114	-0.123	0.142	0.048	0	-0.248	-0.248
DL5	0.130	0.166	0.178	-0.224	-0.110	-0.248	0	0.116
DL6	0.157	0.228	0.213	-0.238	-0.127	-0.248	0.116	0

S1 (g) Table. Observed covariances (lower triangle) and correlations (upper triangle)

	SS1	SS2	SS3	SS4	SS5	SS6
SS1	1	0.575	0.566	0.532	0.591	0.536
SS2	0.575	1	0.829	0.784	0.721	0.821
SS3	0.566	0.829	1	0.841	0.753	0.819
SS4	0.532	0.784	0.841	1	0.764	0.810
SS5	0.591	0.721	0.753	0.764	1	0.833
SS6	0.536	0.821	0.819	0.810	0.833	1

S1(h) Table. Residual covariances (lower triangle) and correlations (upper triangle)

	SS1	SS2	SS3	SS4	SS5	SS6
SS1	0	0.019	-0.008	-0.029	0.050	-0.041
SS2	0.019	0	0.017	-0.010	-0.045	0.004
SS3	-0.008	0.017	0	0.022	-0.037	-0.024
SS4	-0.029	-0.010	0.022	0	-0.009	-0.014
SS5	0.050	-0.045	-0.037	-0.009	0	0.037
SS6	-0.041	0.004	-0.024	-0.014	0.037	0

S1(i) Table. Observed covariances (lower triangle) and correlations (upper triangle)

	WFCt1	WFCt2	WFCs1	WFCs2	WFCb1	WFCb2
WFCt1	1	0.557	0.841	0.538	0.695	0.517
WFCt2	0.557	1	0.608	0.789	0.718	0.674
WFCs1	0.841	0.608	1	0.601	0.780	0.554
WFCs2	0.538	0.789	0.601	1	0.708	0.781
WFCb1	0.695	0.718	0.78	0.708	1	0.659
WFCb2	0.517	0.674	0.554	0.781	0.659	1

S1(j) Table. Residual covariances (lower triangle) and correlations (upper triangle)

	WFCt1	WFCt2	WFCs1	WFCs2	WFCb1	WFCb2
WFCt1	0	-0.139	0.077	-0.198	-0.021	-0.156
WFCt2	-0.139	0	-0.132	0.076	0.024	0.021
WFCs1	0.077	-0.132	0	-0.183	0.018	-0.163
WFCs2	-0.198	0.076	-0.1828	0	-0.027	0.091
WFCb1	-0.021	0.024	0.0177	-0.027	0	-0.014
WFCb2	-0.156	0.021	-0.163	0.091	-0.014	0
