# Supplementary material for: Exploring the link between work-related psychosocial factors and professional quality of life among ethiopian healthcare workers: Insights from structural equation modelling analyses
Source: PLoS One. 2025 Mar 26;20(3):e0319870. doi: 10.1371/journal.pone.0319870 (PMC11940713; doi:10.1371/journal.pone.0319870)
Supplement: S2 Table — (PDF) [file pone.0319870.s002.pdf]

S2 (a) Table. Separate measurement model, reliability, and validity indices for among health workers in central and southern Ethiopia, 2023.

				95% Confidence intervals			95% Confidence intervals					Reliability , and validity Statistics	
Latent	Observed	Estimate	SE	Lower	Upper	β	Lower	Upper	z	p	Communality (R2)	Ordinal Cronbach's alpha	Average Variance Extracted (AVE)
JD	JD1	1.000	0.000	1.000	1.000	0.871	0.849	0.894			0.759	0.896	0.745
	JD2	1.047	0.019	1.009	1.085	0.912	0.895	0.930	53.8	< .001	0.832		
	JD3	0.920	0.016	0.888	0.951	0.801	0.778	0.825	56.4	< .001	0.642		
DL	DL2	1.000	0.000	1.000	1.000	0.605	0.568	0.642			0.366	0.795	0.532
	DL3	1.113	0.041	1.032	1.193	0.673	0.640	0.707	27.1	< .001	0.453		
	DL4	1.128	0.040	1.050	1.207	0.683	0.650	0.716	28.2	< .001	0.466		
	DL5	1.409	0.047	1.317	1.501	0.853	0.828	0.877	29.9	< .001	0.727		
	DL6	1.328	0.046	1.238	1.418	0.803	0.777	0.830	28.9	< .001	0.645		
SS	SS1	1.000	0.000	1.000	1.000	0.626	0.595	0.658			0.392	0.939	0.735
	SS2	1.420	0.035	1.350	1.480	0.887	0.874	0.900	40.4	< .001	0.787		
	SS3	1.460	0.038	1.390	1.540	0.915	0.903	0.927	38.3	< .001	0.837		
	SS4	1.430	0.036	1.360	1.500	0.895	0.883	0.907	39.9	< .001	0.801		
	SS5	1.380	0.035	1.310	1.450	0.864	0.849	0.879	39.4	< .001	0.746		
	SS6	1.470	0.037	1.400	1.540	0.921	0.912	0.930	39.6	< .001	0.849		
JE	ERI1	1.000	0.000	1.000	1.000	0.856	0.808	0.905			0.733	0.775	0.542
	ERI2	0.793	0.043	0.709	0.878	0.679	0.631	0.727	18.4	< .001	0.461		
	ERI3	0.766	0.042	0.684	0.848	0.656	0.606	0.706	18.3	< .001	0.430		
JR	ERI4	1.000	0.000	1.000	1.000	0.645	0.614	0.677			0.416	0.925	0.662
	ERI5	1.135	0.032	1.073	1.197	0.732	0.708	0.756	35.9	< .001	0.536		
	ERI6	1.277	0.033	1.213	1.341	0.824	0.805	0.843	39.2	< .001	0.679		
	ERI7	1.171	0.034	1.104	1.237	0.755	0.731	0.780	34.4	< .001	0.571		
	ERI8	1.364	0.034	1.298	1.431	0.880	0.867	0.894	40.2	< .001	0.775		
	ERI9	1.464	0.037	1.391	1.536	0.944	0.935	0.954	39.5	< .001	0.892		

	ERI10	1.359	0.035	1.290	1.427	0.877	0.862	0.892	38.9	< .001	0.768		
WFC	WFCt1	1.000	0.000	1.000	1.000	0.847	0.830	0.864			0.718	0.923	0.718
	WFCt2	0.968	0.012	0.944	0.992	0.820	0.802	0.838	79.6	< .001	0.673		
	WFCs1	1.064	0.014	1.037	1.091	0.902	0.888	0.915	78.3	< .001	0.813		
	WFCs2	1.025	0.012	1.002	1.049	0.869	0.854	0.884	85.3	< .001	0.755		
	WFCb1	0.998	0.011	0.978	1.019	0.846	0.830	0.861	95.5	< .001	0.715		
	WFCb2	0.938	0.014	0.911	0.966	0.795	0.774	0.816	67.4	< .001	0.632		
BO	BO1	1.000	0.000	1.000	1.000	0.586	0.546	0.625			0.343	0.695	0.534
	BO2	1.257	0.046	1.167	1.348	0.736	0.710	0.763	27.30	< .001	0.542		
	BO3	1.446	0.050	1.348	1.544	0.847	0.826	0.868	29.00	< .001	0.717		
CF	CF1	1.000	0.000	1.000	1.000	0.682	0.640	0.723			0.465	0.717	0.501
	CF2	0.921	0.034	0.854	0.987	0.628	0.595	0.660	27.20	< .001	0.394		
	CF3	1.179	0.038	1.104	1.254	0.804	0.776	0.832	30.90	< .001	0.646		
CS	CS1	1.000	0.000	1.000	1.000	0.899	0.874	0.923			0.807	0.850	0.670
	CS2	0.925	0.024	0.878	0.971	0.831	0.805	0.857	39.00	< .001	0.690		
	CS3	0.797	0.019	0.759	0.835	0.716	0.688	0.744	41.20	< .001	0.512		

Note: BO, Burnout Syndrome; CF, Compassion Fatigue; CS, Compassion Satisfaction; JD , Job demand; DL, Decision latitude; SS, Social Support; JE, Job effort; JR, Job Reward, WFC, Work Family Conflict; ERI, Effort Reward Imbalance; WFCt, Work Family Conflict time base; WFCs, Work Family Conflict Strain based; WFCb, Work Family Conflict Behaviour Based

S2(b) Table. Summary of additional information for CFA of the measurement model among health workers in central and southern Ethiopia, 2023.

Model aspects	Validated measures based on previous theoretical models								
	Job demand control		Social Support (SS)	Effort-Reward Imbalance		Work-family Conflict (WFC)	Professional Quality of Life (PQoL)		
	Job demand (JD)	Decision latitude (DL)		Job Effort (JE)	Job Reward (JR)		BO	CF	CS
Model Estimation Method	Diagonally weighted least squares (DWLS)		DWLS	DWLS		DWLS	DWLS		
Optimization Method	Nonlinear minimization with box constraints (NLMINB)		NLMINB	NLMINB		NLMINB	NLMINB		
Free parameters & Iterations	59 free parameters & 38 iterations		24 free parameters & 18 iteration	42 free parameters and 29 iterations		30 free parameters & 17 iterations	48 free parameters and 35 iterations		
Model dimensions*	CFA full model for 3 subscales: JD, DL & SS		Treated as a unidimensional	CFA full model for 2 subscales: JE & JR		Treated as a unidimensional	CFA model fit for 3 subscales: BO, CF, &CS		
Overall Model Fit	$\chi^2(74) = 1574$, $p < 0.001$		$\chi^2(9) = 81.2$, $p < 0.001$	$\chi^2(34) = 350$, $p < 0.001$		$\chi^2(9) = 645$, $p < 0.001$	$\chi^2(24) = 794$, $p < 0.001$		
Model Fit Indices	SRMR = 0.081, RMSEA = 0.123, AGFI = 0.913, GFI = 0.968, CFI = 0.956, TLI = 0.935		SRMR = 0.018, RMSEA = 0.149, AGFI = 0.997, GFI = 0.999, CFI = 0.965, TLI = 0.942	SRMR = 0.035, RMSEA = 0.081, AGFI = 0.990, GFI = 0.995, CFI = 0.995, TLI = 0.993		SRMR = 0.064, RMSEA = 0.346, CFI = 0.985, TLI = 0.975, GFI=0.988, AGFI=0.946	SRMR = 0.077, RMSEA = 0.190, CFI = 0.963, TLI = 0.945, GFI=0.978, AGFI=0.935		
Communality (R^2)	The variances in DL2, DL3, and DL4 accounted for 36.6%, 0.45.3%, and 0.46.6% explained by DL subscale, and all other JD items and DL items are explained by their respective subscales ranging from 64.2% to 83.2%.		The variances in SS1 accounted for 39.2% explained by SS, the rest items are explained from 74. 6% for SS5 and 84.9% for SS6.	The minimum variance in JE item accounted for 43.0 % (ERI3) explained by JE subscale, and 41.6% (ERI 4) explained by JR subscale of ERI		The variance explained by WFC was 63.2% WFCb2 and the maximum variance was 81.3% for WFCs1	The minimum variance accounted for BO1, CF2, and CS3 were 34.3 % , 39.2%, and 51.2% fro BO1, CF2 and CS3, respectively explained by each subscale		

Standardised factor loading	The minimum factor load was 0.605 for DL2 and the highest was 0.912 for JD4 suggesting all have acceptable factor load (>0.50)	The minimum factor load was 0.626 for SS1 and the rest were 0.864 above suggesting an acceptable factor load (>0.50)	The minimum factor load was 0.645 for ERI4 and the highest was 0.944 for ER9 suggesting all items have acceptable factor load (>0.50)	The minimum factor load was 0.795 for WFCb2 and the maximum was 0.902 for WFCs1 suggesting an acceptable factor load (>0.50)	The minimum factor load was 0.586 for BO1 and the highest was 0.899 for CS1 suggesting all items have acceptable factor load (>0.50)
Observed covariances and correlations	The observed correlations between items within a JD subscale range from 0.690 (between JD1, and JD3) to 0.793 (between JD1 and JD2), and within A DL it ranges from 0.248 (between DL2 & DL6) to 0.801 (between DL5 and DL6) suggesting acceptable range (above 0.30). All observed correlations have expected direction (All the correlations between the job demand and decision latitude items have significant negative correlations)	The observed correlations ranged from 0.532 (between SS1 and SS4) to 0.833 (between SS5 and SS6), and all correlations were reasonable.	The observed correlations between items within a JE subscale range from 0.497 (between ERI2 and ERI3) to 0.571 (between ERI1 and ERI2), and within a JR it ranges from 0.446 (between ERI5 & ERI7) to 0.865(between ERI9 & ERI10) suggesting acceptable range (above 0.30). All observed correlations have expected directions (All the correlations between the job effort and reward items have significant negative correlations)	The observed correlations ranged from 0.517 (between WFCt1 and WFCs1) to 0.841 (between WFCt1 and WFCs1), and all correlations were reasonable.	The observed correlations between items within a PQoL subscale range from 0.300 (between BO1 and BO2)) to 0.694 (between BO2 and BO3) , and within a CF it ranges from 0.345 (between CF2 & CF1) to 0.441(between CF1 & CF3), 0.572 (CS3 and CS2) to 0.762 (CS1 and CS2) suggesting acceptable range (above 0.30). All observed correlations have expected directions (All the correlations between the job effort and reward items have significant negative correlations)
Residual covariances and correlations	There are residual correlations and covariances were between -0.238 and 0.228	There are residual correlations and covariances ranging from 0.4% (between SS2 and SS6) to 5% (between SS1 & SS5)	There are residual correlations between -0.005 and 0.130	Residual correlations and covariances are ranging from 1.7% (between WFCs1 and WFCb1) to 19.81% (between WFCt1 & WFCS2)	Residual correlations and covariances are ranging from 0.2% (between BO3 and CF2) to 25.7% (between CF1 & BO1)

* CFA full model was performed based on previously validated respective measurements and theory
