# Supplementary material for: Exploring the link between work-related psychosocial factors and professional quality of life among ethiopian healthcare workers: Insights from structural equation modelling analyses
Source: PLoS One. 2025 Mar 26;20(3):e0319870. doi: 10.1371/journal.pone.0319870 (PMC11940713; doi:10.1371/journal.pone.0319870)
Supplement: S3 Table — (PDF) [file pone.0319870.s003.Pdf]

S3(a) Table. Observed covariances (lower triangle) and correlations (upper triangle) between items of constructs in the study of work-related psychosocial factors, and PQoL among healthcare workers in central and southern Ethiopia, 2023.

	JD1	JD2	JD3	DL2	DL3	DL4	DL5	DL6	ERI1	ERI2	ERI3	ERI4	ERI5	ERI6	ERI7	ERI8	ERI9	ERI10
JD1	1	0.794	0.690	-0.351	-0.454	-0.459	-0.278	-0.230	0.103	0.033	0.035	-0.123	-0.203	-0.205	-0.186	-0.197	-0.254	-0.251
JD2	0.794	1	0.739	-0.399	-0.433	-0.456	-0.261	-0.174	0.084	0.061	0.053	-0.160	-0.204	-0.244	-0.223	-0.232	-0.260	-0.272
JD3	0.690	0.739	1	-0.406	-0.377	-0.424	-0.198	-0.143	0.072	0.049	-0.022	-0.139	-0.129	-0.183	-0.167	-0.157	-0.206	-0.211
DL2	-0.351	-0.399	-0.406	1	0.449	0.557	0.294	0.252	-0.178	-0.058	-0.076	0.207	0.220	0.251	0.233	0.240	0.239	0.272
DL3	-0.454	-0.433	-0.377	0.449	1	0.510	0.464	0.416	-0.140	-0.025	-0.028	0.132	0.179	0.195	0.188	0.200	0.253	0.205
DL4	-0.459	-0.456	-0.424	0.557	0.510	1	0.335	0.301	-0.123	-0.078	-0.049	0.216	0.276	0.287	0.300	0.297	0.358	0.313
DL5	-0.278	-0.261	-0.199	0.294	0.464	0.335	1	0.802	-0.187	-0.041	-0.105	0.101	0.169	0.118	0.146	0.157	0.219	0.194
DL6	-0.230	-0.174	-0.143	0.252	0.416	0.301	0.802	1	-0.162	-0.039	-0.080	0.102	0.132	0.087	0.106	0.108	0.170	0.146
ERI1	0.103	0.084	0.072	-0.178	-0.140	-0.123	-0.187	-0.162	1	0.570	0.535	-0.194	-0.321	-0.227	-0.240	-0.292	-0.306	-0.273
ERI2	0.033	0.061	0.049	-0.058	-0.025	-0.078	-0.041	-0.039	0.570	1	0.496	-0.138	-0.214	-0.184	-0.232	-0.187	-0.182	-0.115
ERI3	0.035	0.053	-0.022	-0.076	-0.028	-0.049	-0.105	-0.080	0.535	0.496	1	-0.127	-0.209	-0.184	-0.190	-0.176	-0.198	-0.177
ERI4	-0.123	-0.160	-0.139	0.207	0.132	0.216	0.101	0.102	-0.194	-0.138	-0.127	1	0.483	0.623	0.445	0.599	0.509	0.510
ERI5	-0.203	-0.204	-0.129	0.220	0.179	0.276	0.169	0.132	-0.321	-0.214	-0.209	0.483	1	0.573	0.685	0.561	0.687	0.572
ERI6	-0.205	-0.244	-0.183	0.251	0.195	0.287	0.118	0.087	-0.227	-0.184	-0.184	0.623	0.573	1	0.637	0.771	0.708	0.678
ERI7	-0.186	-0.223	-0.167	0.233	0.188	0.300	0.146	0.106	-0.240	-0.232	-0.190	0.445	0.685	0.637	1	0.655	0.651	0.599
ERI8	-0.197	-0.232	-0.157	0.240	0.200	0.297	0.157	0.108	-0.292	-0.187	-0.176	0.599	0.561	0.772	0.655	1	0.825	0.764
ERI9	-0.254	-0.260	-0.206	0.239	0.253	0.358	0.219	0.170	-0.306	-0.182	-0.198	0.509	0.687	0.708	0.651	0.825	1	0.865
ERI10	-0.251	-0.272	-0.211	0.272	0.205	0.313	0.194	0.146	-0.273	-0.115	-0.178	0.510	0.572	0.678	0.599	0.764	0.865	1
WFCt1	0.370	0.391	0.366	-0.259	-0.315	-0.385	-0.259	-0.208	0.216	0.115	0.108	-0.255	-0.313	-0.286	-0.318	-0.349	-0.387	-0.362
WFCt2	0.283	0.343	0.274	-0.337	-0.289	-0.340	-0.177	-0.141	0.163	0.126	0.078	-0.197	-0.291	-0.311	-0.305	-0.319	-0.334	-0.316
WFCs1	0.384	0.414	0.365	-0.309	-0.368	-0.440	-0.255	-0.227	0.185	0.120	0.087	-0.240	-0.324	-0.300	-0.351	-0.368	-0.411	-0.369
WFCs2	0.280	0.353	0.329	-0.395	-0.253	-0.366	-0.159	-0.135	0.150	0.137	0.091	-0.240	-0.321	-0.316	-0.331	-0.331	-0.352	-0.331
WFCb1	0.296	0.362	0.312	-0.353	-0.313	-0.405	-0.240	-0.199	0.178	0.141	0.077	-0.238	-0.310	-0.325	-0.312	-0.334	-0.379	-0.353
WFCb2	0.263	0.298	0.255	-0.385	-0.269	-0.413	-0.158	-0.127	0.187	0.147	0.107	-0.242	-0.338	-0.304	-0.331	-0.335	-0.360	-0.339
BO1	0.243	0.229	0.207	-0.181	-0.191	-0.306	-0.151	-0.114	0.197	0.146	0.095	-0.266	-0.345	-0.280	-0.317	-0.352	-0.395	-0.366
BO2	0.374	0.351	0.292	-0.328	-0.259	-0.420	-0.214	-0.179	0.191	0.076	0.071	-0.181	-0.270	-0.227	-0.264	-0.247	-0.305	-0.284
BO3	0.329	0.357	0.335	-0.344	-0.229	-0.430	-0.163	-0.113	0.159	0.113	0.062	-0.201	-0.243	-0.242	-0.253	-0.261	-0.298	-0.300
CF1	0.256	0.258	0.192	-0.287	-0.246	-0.357	-0.155	-0.124	0.205	0.161	0.089	-0.295	-0.379	-0.361	-0.356	-0.375	-0.415	-0.411
CF2	0.259	0.219	0.196	-0.231	-0.226	-0.336	-0.172	-0.165	0.175	0.094	0.036	-0.134	-0.187	-0.200	-0.200	-0.239	-0.272	-0.247
CF3	0.254	0.269	0.275	-0.338	-0.217	-0.431	-0.185	-0.132	0.234	0.138	0.091	-0.204	-0.301	-0.321	-0.326	-0.336	-0.358	-0.346
CS1	-0.156	-0.180	-0.151	0.310	0.200	0.341	0.150	0.165	-0.099	-0.062	-0.064	0.194	0.200	0.215	0.179	0.185	0.214	0.180
CS2	-0.161	-0.157	-0.124	0.290	0.194	0.356	0.174	0.193	-0.152	-0.113	-0.112	0.174	0.218	0.230	0.198	0.221	0.246	0.208
CS3	-0.251	-0.204	-0.196	0.279	0.186	0.373	0.191	0.173	-0.179	-0.107	-0.078	0.209	0.215	0.193	0.194	0.223	0.276	0.228
SS1	-0.104	-0.171	-0.103	0.055	0.034	0.094	-0.042	-0.088	-0.079	-0.079	-0.090	0.121	0.091	0.146	0.122	0.114	0.107	0.101
SS2	-0.124	-0.169	-0.143	0.138	0.079	0.274	-0.015	0.034	-4.22e−4	-0.051	-0.020	0.111	0.154	0.123	0.135	0.112	0.120	0.128
SS3	-0.114	-0.160	-0.110	0.145	0.163	0.292	0.006	0.045	-0.050	-0.078	-0.043	0.133	0.201	0.163	0.206	0.174	0.184	0.176
SS4	-0.126	-0.166	-0.132	0.134	0.116	0.224	0.027	0.059	-0.042	-0.080	-0.067	0.140	0.164	0.109	0.140	0.119	0.137	0.118
SS5	-0.143	-0.207	-0.174	0.231	0.103	0.293	-0.009	-0.018	-0.043	-0.049	-0.047	0.172	0.218	0.210	0.186	0.201	0.217	0.200
SS6	-0.138	-0.165	-0.178	0.151	0.077	0.251	-0.006	0.040	-0.003	-0.066	-0.036	0.134	0.169	0.131	0.163	0.139	0.135	0.138

	WFCt1	WFCt2	WFCs1	WFCs2	WFCb1	WFCb2	BO1	BO2	BO3	CF1	CF2	CF3	CS1	CS2	CS3	SS1	SS2	SS3	SS4	SS5	SS6
JD1	0.370	0.283	0.384	0.280	0.296	0.263	0.243	0.374	0.329	0.256	0.259	0.254	-0.156	-0.161	-0.251	-0.104	-0.124	-0.114	-0.126	-0.143	-0.138
JD2	0.391	0.343	0.414	0.353	0.362	0.298	0.229	0.351	0.357	0.258	0.219	0.269	-0.180	-0.157	-0.204	-0.171	-0.169	-0.160	-0.166	-0.207	-0.165
JD3	0.366	0.274	0.365	0.329	0.312	0.255	0.207	0.292	0.335	0.192	0.196	0.275	-0.151	-0.124	-0.196	-0.103	-0.143	-0.110	-0.132	-0.174	-0.178
DL2	-0.259	-0.337	-0.309	-0.395	-0.353	-0.385	-0.181	-0.328	-0.344	-0.287	-0.231	-0.338	0.310	0.290	0.279	0.055	0.138	0.145	0.134	0.231	0.151
DL3	-0.315	-0.289	-0.368	-0.253	-0.313	-0.269	-0.191	-0.259	-0.229	-0.246	-0.226	-0.217	0.200	0.194	0.186	0.034	0.079	0.163	0.116	0.103	0.077
DL4	-0.385	-0.340	-0.440	-0.366	-0.405	-0.413	-0.306	-0.420	-0.430	-0.357	-0.336	-0.431	0.341	0.356	0.373	0.094	0.274	0.291	0.224	0.293	0.251
DL5	-0.259	-0.177	-0.255	-0.159	-0.240	-0.158	-0.151	-0.214	-0.163	-0.155	-0.172	-0.185	0.150	0.174	0.191	-0.042	-0.015	0.006	0.027	-0.009	-0.006
DL6	-0.208	-0.141	-0.227	-0.135	-0.199	-0.127	-0.114	-0.179	-0.113	-0.124	-0.165	-0.132	0.165	0.193	0.173	-0.088	0.034	0.045	0.059	-0.018	0.040
ERI1	0.216	0.163	0.185	0.150	0.178	0.187	0.197	0.191	0.159	0.205	0.175	0.234	-0.099	-0.152	-0.179	-0.080	-4.22e−4	-0.050	-0.042	-0.043	-0.003
ERI2	0.115	0.126	0.120	0.137	0.141	0.147	0.146	0.076	0.113	0.161	0.094	0.138	-0.062	-0.113	-0.107	-0.079	-0.051	-0.078	-0.080	-0.049	-0.066
ERI3	0.108	0.078	0.087	0.091	0.077	0.107	0.095	0.071	0.062	0.089	0.036	0.091	-0.064	-0.112	-0.078	-0.090	-0.020	-0.043	-0.067	-0.047	-0.036
ERI4	-0.255	-0.197	-0.240	-0.240	-0.238	-0.242	-0.266	-0.181	-0.201	-0.295	-0.134	-0.204	0.194	0.174	0.209	0.121	0.111	0.133	0.140	0.172	0.134
ERI5	-0.313	-0.291	-0.324	-0.321	-0.310	-0.337	-0.345	-0.270	-0.243	-0.379	-0.187	-0.301	0.200	0.218	0.215	0.091	0.154	0.201	0.164	0.218	0.169
ERI6	-0.286	-0.311	-0.300	-0.316	-0.325	-0.304	-0.280	-0.227	-0.242	-0.361	-0.200	-0.321	0.215	0.230	0.193	0.146	0.123	0.163	0.109	0.210	0.131
ERI7	-0.318	-0.305	-0.351	-0.331	-0.312	-0.331	-0.317	-0.264	-0.253	-0.356	-0.200	-0.326	0.179	0.198	0.194	0.122	0.136	0.206	0.140	0.186	0.163
ERI8	-0.349	-0.319	-0.368	-0.331	-0.334	-0.335	-0.352	-0.247	-0.261	-0.375	-0.239	-0.336	0.185	0.221	0.223	0.114	0.112	0.174	0.119	0.201	0.139
ERI9	-0.387	-0.334	-0.411	-0.352	-0.379	-0.360	-0.395	-0.305	-0.298	-0.415	-0.272	-0.358	0.214	0.246	0.276	0.107	0.121	0.184	0.137	0.217	0.135
ERI10	-0.362	-0.316	-0.369	-0.331	-0.353	-0.339	-0.366	-0.284	-0.300	-0.411	-0.247	-0.346	0.180	0.208	0.228	0.101	0.128	0.176	0.118	0.200	0.138
WFCt1	1	0.555	0.841	0.540	0.694	0.515	0.346	0.384	0.392	0.379	0.287	0.320	-0.216	-0.205	-0.326	-0.172	-0.168	-0.216	-0.205	-0.229	-0.192
WFCt2	0.555	1	0.606	0.791	0.716	0.671	0.309	0.265	0.392	0.409	0.301	0.374	-0.267	-0.224	-0.298	-0.121	-0.128	-0.174	-0.173	-0.208	-0.136
WFCs1	0.841	0.606	1	0.601	0.779	0.551	0.399	0.410	0.436	0.441	0.358	0.376	-0.241	-0.233	-0.334	-0.132	-0.163	-0.191	-0.190	-0.226	-0.169
WFCs2	0.540	0.791	0.601	1	0.709	0.784	0.342	0.266	0.393	0.457	0.310	0.403	-0.332	-0.281	-0.355	-0.114	-0.212	-0.243	-0.231	-0.263	-0.227
WFCb1	0.694	0.716	0.779	0.709	1	0.656	0.387	0.331	0.406	0.456	0.319	0.361	-0.279	-0.282	-0.311	-0.153	-0.174	-0.208	-0.187	-0.242	-0.162
WFCb2	0.515	0.671	0.551	0.784	0.656	1	0.414	0.324	0.399	0.483	0.356	0.416	-0.312	-0.298	-0.372	-0.138	-0.204	-0.248	-0.209	-0.276	-0.216
BO1	0.346	0.309	0.399	0.342	0.387	0.414	1	0.300	0.306	0.676	0.272	0.298	-0.204	-0.212	-0.307	-0.195	-0.241	-0.259	-0.226	-0.330	-0.236
BO2	0.384	0.265	0.410	0.266	0.331	0.324	0.300	1	0.693	0.341	0.545	0.602	-0.110	-0.074	-0.227	-0.193	-0.180	-0.248	-0.219	-0.284	-0.230
BO3	0.392	0.392	0.436	0.393	0.406	0.399	0.306	0.693	1	0.368	0.558	0.747	-0.197	-0.162	-0.208	-0.186	-0.162	-0.197	-0.203	-0.293	-0.210
CF1	0.379	0.409	0.441	0.457	0.456	0.482	0.676	0.341	0.368	1	0.347	0.442	-0.310	-0.307	-0.366	-0.147	-0.266	-0.301	-0.301	-0.342	-0.272
CF2	0.287	0.301	0.358	0.310	0.319	0.356	0.272	0.545	0.558	0.347	1	0.587	-0.063	-0.062	-0.093	-0.134	-0.123	-0.157	-0.127	-0.174	-0.113
CF3	0.320	0.374	0.376	0.403	0.361	0.416	0.298	0.602	0.747	0.442	0.587	1	-0.257	-0.245	-0.240	-0.117	-0.200	-0.219	-0.202	-0.260	-0.196
CS1	-0.216	-0.267	-0.241	-0.332	-0.279	-0.312	-0.204	-0.110	-0.197	-0.310	-0.063	-0.257	1	0.761	0.623	0.094	0.293	0.296	0.276	0.302	0.303
CS2	-0.205	-0.224	-0.233	-0.281	-0.282	-0.298	-0.212	-0.074	-0.162	-0.307	-0.062	-0.245	0.761	1	0.571	0.134	0.318	0.321	0.278	0.336	0.330
CS3	-0.326	-0.298	-0.334	-0.355	-0.311	-0.372	-0.307	-0.227	-0.208	-0.366	-0.093	-0.240	0.623	0.571	1	0.236	0.307	0.326	0.278	0.340	0.323
SS1	-0.172	-0.121	-0.132	-0.114	-0.153	-0.138	-0.195	-0.193	-0.186	-0.147	-0.134	-0.117	0.094	0.134	0.236	1	0.574	0.566	0.532	0.589	0.534
SS2	-0.168	-0.128	-0.163	-0.212	-0.174	-0.204	-0.241	-0.180	-0.162	-0.266	-0.123	-0.200	0.293	0.318	0.307	0.574	1	0.829	0.785	0.720	0.821
SS3	-0.216	-0.174	-0.191	-0.243	-0.208	-0.248	-0.259	-0.248	-0.198	-0.301	-0.157	-0.219	0.296	0.321	0.326	0.566	0.829	1	0.841	0.754	0.820
SS4	-0.205	-0.173	-0.190	-0.231	-0.187	-0.209	-0.226	-0.219	-0.203	-0.301	-0.127	-0.202	0.276	0.278	0.278	0.532	0.785	0.841	1	0.765	0.812
SS5	-0.229	-0.208	-0.226	-0.263	-0.242	-0.276	-0.330	-0.284	-0.293	-0.342	-0.174	-0.260	0.302	0.336	0.340	0.589	0.721	0.754	0.765	1	0.832
SS6	-0.192	-0.136	-0.169	-0.227	-0.162	-0.216	-0.236	-0.230	-0.210	-0.272	-0.113	-0.196	0.303	0.330	0.323	0.534	0.821	0.820	0.812	0.832	1

S3(b) Table. Residual covariances (lower triangle) and correlations (upper triangle) between items of constructs in the study of work-related psychosocial factors, and PQoL among healthcare workers in central and southern Ethiopia, 2023.

	JD1	JD2	JD3	DL2	DL3	DL4	DL5	DL6	ERI1	ERI2	ERI3	ERI4	ERI5	ERI6	ERI7	ERI8	ERI9	ERI10
JD1	0	-8.56e−4	-0.002	-0.016	-0.138	-0.051	0.081	0.107	0.032	-0.018	-0.013	0.038	-0.016	0.000	0.006	0.022	-0.017	-0.031
JD2	-8.56[illegible]	0	0.002	-0.043	-0.096	-0.021	0.122	0.185	0.009	0.006	0.003	0.010	-0.005	-0.026	-0.018	0.002	-0.007	-0.038
JD3	-0.002	0.002	0	-0.096	-0.084	-0.045	0.134	0.169	0.007	0.002	-0.066	0.010	0.044	0.008	0.011	0.046	0.014	-0.007
DL2	-0.016	-0.043	-0.096	0	-0.031	-0.064	-0.252	-0.261	-0.061	0.026	0.002	0.052	0.040	0.053	0.047	0.028	0.010	0.059
DL3	-0.138	-0.096	-0.084	-0.031	0	-0.076	-0.051	-0.067	-0.030	0.055	0.046	-0.014	0.008	0.008	0.013	-5.86e−4	0.036	0.004
DL4	-0.051	-0.021	-0.045	-0.064	-0.076	0	-0.331	-0.324	0.019	0.025	0.047	0.027	0.056	0.045	0.073	0.038	0.079	0.053
DL5	0.081	0.122	0.134	-0.252	-0.051	-0.331	0	0.252	-0.062	0.049	-0.020	-0.065	-0.025	-0.095	-0.053	-0.071	-0.027	-0.034
DL6	0.107	0.185	0.169	-0.261	-0.067	-0.324	0.252	0	-0.045	0.046	-0.001	-0.054	-0.050	-0.112	-0.080	-0.106	-0.060	-0.069
ERI1	0.032	0.009	0.007	-0.061	-0.030	0.019	-0.062	-0.045	0	-0.030	-0.023	0.012	-0.081	0.037	0.006	-0.010	-0.002	0.010
ERI2	-0.018	0.006	0.002	0.026	0.055	0.025	0.050	0.046	-0.030	0	0.091	0.011	-0.040	0.007	-0.054	0.018	0.038	0.090
ERI3	-0.013	0.003	-0.066	0.002	0.046	0.047	-0.020	-0.001	-0.023	0.091	0	0.012	-0.048	-0.007	-0.024	0.014	0.007	0.013
ERI4	0.038	0.010	0.010	0.052	-0.014	0.027	-0.065	-0.054	0.012	0.011	0.012	0	0.007	0.100	-0.044	0.040	-0.094	-0.050
ERI5	-0.016	-0.005	0.044	0.040	0.008	0.056	-0.025	-0.050	-0.081	-0.040	-0.048	0.007	0	-0.037	0.115	-0.091	-0.017	-0.081
ERI6	0.000	-0.026	0.008	0.053	0.008	0.045	-0.095	-0.112	0.037	0.007	-0.007	0.100	-0.037	0	0.011	0.056	-0.065	-0.040
ERI7	0.006	-0.018	0.011	0.047	0.013	0.073	-0.054	-0.080	0.006	-0.054	-0.024	-0.044	0.115	0.011	0	-0.015	-0.071	-0.073
ERI8	0.022	0.002	0.046	0.028	-5.86e−4	0.038	-0.071	-0.106	-0.010	0.018	0.014	0.040	-0.091	0.056	-0.015	0	-8.70e−4	-0.004
ERI9	-0.017	-0.007	0.014	0.010	0.036	0.079	-0.027	-0.060	-0.002	0.038	0.007	-0.094	-0.017	-0.065	-0.071	-8.70e−4	0	0.036
ERI10	-0.031	-0.038	-0.007	0.059	0.004	0.053	-0.034	-0.069	0.010	0.090	0.013	-0.050	-0.081	-0.040	-0.073	-0.004	0.036	0
WFCt1	0.036	0.035	0.056	0.019	-0.052	-0.045	0.040	0.073	0.046	-0.009	-0.006	-0.004	-0.019	0.037	-0.017	-0.004	-0.014	-0.016
WFCt2	-0.036	0.003	-0.022	-0.071	-0.038	-0.016	0.108	0.127	0.000	0.008	-0.031	0.044	-0.011	-0.003	-0.016	0.011	0.022	0.014
WFCs1	0.027	0.034	0.035	-0.011	-0.087	-0.077	0.064	0.073	0.003	-0.012	-0.036	0.029	-0.010	0.044	-0.029	0.001	-0.014	0.000
WFCs2	-0.061	-0.011	0.013	-0.110	0.016	-0.019	0.147	0.152	-0.024	0.011	-0.026	0.017	-0.021	0.014	-0.022	0.021	0.028	0.022
WFCb1	-0.038	0.006	0.002	-0.074	-0.049	-0.064	0.059	0.082	0.007	0.018	-0.038	0.015	-0.015	-0.002	-0.010	0.012	-0.006	-0.006
WFCb2	-0.061	-0.047	-0.044	-0.116	-0.014	-0.084	0.131	0.144	0.022	0.027	-0.004	0.002	-0.053	0.009	-0.039	-5.38e−4	0.000	-0.005
BO1	-0.038	-0.070	-0.054	0.045	0.022	-0.031	0.091	0.113	0.053	0.042	-0.002	-0.066	-0.112	-0.024	-0.077	-0.078	-0.099	-0.091
BO2	0.067	0.024	0.007	-0.082	-0.026	-0.120	0.050	0.069	0.034	-0.038	-0.035	0.037	-0.015	0.053	-0.003	0.052	0.017	0.016
BO3	-0.010	-0.005	0.020	-0.071	0.028	-0.098	0.130	0.162	-0.015	-0.013	-0.055	0.041	0.039	0.068	0.036	0.070	0.060	0.032
CF1	-0.006	-0.020	-0.050	-0.024	0.002	-0.036	0.127	0.141	0.015	0.023	-0.040	-0.040	-0.082	-0.035	-0.051	-0.026	-0.038	-0.061
CF2	0.053	-1.71e−4	0.005	-0.024	-0.031	-0.083	0.051	0.044	0.025	-0.015	-0.065	0.067	0.048	0.058	0.041	0.036	0.025	0.029
CF3	-0.010	-0.013	0.030	-0.072	0.034	-0.106	0.101	0.136	0.041	-0.002	-0.039	0.053	-6.40e−4	0.009	-0.018	0.017	0.023	0.008
CS1	0.025	0.012	0.016	0.069	-0.027	0.047	-0.108	-0.078	0.040	0.038	0.029	0.026	0.004	0.000	-0.022	-0.045	-0.035	-0.051
CS2	0.017	0.033	0.041	0.053	-0.030	0.067	-0.081	-0.045	-0.015	-0.013	-0.020	0.009	0.025	0.018	-2.79e−4	-0.006	0.001	-0.020
CS3	-0.080	-0.022	-0.037	0.050	-0.030	0.094	-0.054	-0.057	-0.047	-0.011	0.011	0.050	0.029	-0.012	0.003	0.005	0.040	0.009
SS1	0.002	-0.059	-0.005	-0.021	-0.038	0.002	-0.124	-0.165	-0.037	-0.048	-0.062	0.037	-0.007	0.038	0.022	-0.001	-0.017	-0.015
SS2	0.027	-0.008	-0.002	0.029	-0.023	0.142	-0.131	-0.075	0.060	-0.007	0.020	-0.009	0.013	-0.031	-0.008	-0.052	-0.057	-0.037
SS3	0.044	0.008	0.036	0.033	0.056	0.154	-0.115	-0.068	0.013	-0.032	-7.85e−4	0.008	0.056	0.003	0.057	0.003	-0.001	0.004
SS4	0.027	-0.003	0.010	0.024	0.013	0.091	-0.091	-0.051	0.019	-0.036	-0.026	0.018	0.022	-0.046	-0.005	-0.047	-0.043	-0.048
SS5	0.008	-0.047	-0.034	0.123	0.001	0.161	-0.125	-0.126	0.017	-0.006	-0.006	0.052	0.079	0.057	0.043	0.037	0.040	0.036
SS6	0.019	0.003	-0.033	0.038	-0.029	0.113	-0.127	-0.074	0.059	-0.020	0.007	0.009	0.023	-0.029	0.014	-0.032	-0.050	-0.033

	WFCt1	WFCt2	WFCs1	WFCs2	WFCb1	WFCb2	BO1	BO2	BO3	CF1	CF2	CF3	CS1	CS2	CS3	SS1	SS2	SS3	SS4	SS5	SS6
JD1	0.036	-0.036	0.027	-0.061	-0.038	-0.061	-0.038	0.067	-0.010	-0.006	0.053	-0.010	0.025	0.017	-0.080	0.002	0.027	0.044	0.027	0.008	0.019
JD2	0.035	0.003	0.034	-0.011	0.006	-0.047	-0.070	0.024	-0.005	-0.020	-1.71e−4	-0.013	0.012	0.033	-0.022	-0.059	-0.008	0.008	-0.003	-0.047	0.003
JD3	0.056	-0.022	0.035	0.013	0.002	-0.044	-0.054	0.007	0.020	-0.050	0.005	0.030	0.016	0.041	-0.037	-0.005	-0.002	0.036	0.010	-0.034	-0.033
DL2	0.019	-0.071	-0.011	-0.110	-0.074	-0.116	0.045	-0.082	-0.071	-0.024	-0.024	-0.072	0.069	0.053	0.050	-0.021	0.029	0.033	0.024	0.123	0.038
DL3	-0.052	-0.038	-0.087	0.016	-0.049	-0.014	0.022	-0.026	0.028	0.002	-0.031	0.034	-0.027	-0.030	-0.030	-0.038	-0.023	0.056	0.013	0.001	-0.029
DL4	-0.045	-0.016	-0.077	-0.019	-0.064	-0.084	-0.031	-0.120	-0.098	-0.036	-0.083	-0.106	0.047	0.067	0.094	0.002	0.142	0.154	0.091	0.161	0.113
DL5	0.040	0.108	0.064	0.147	0.059	0.131	0.091	0.050	0.129	0.127	0.051	0.101	-0.108	-0.081	-0.054	-0.124	-0.131	-0.115	-0.091	-0.125	-0.127
DL6	0.073	0.127	0.073	0.152	0.082	0.144	0.113	0.069	0.162	0.141	0.044	0.136	-0.078	-0.045	-0.057	-0.165	-0.075	-0.068	-0.051	-0.126	-0.074
ERI1	0.046	0.000	0.003	-0.024	0.007	0.022	0.053	0.034	-0.015	0.015	0.025	0.041	0.040	-0.015	-0.047	-0.037	0.060	0.013	0.019	0.017	0.059
ERI2	-0.009	0.008	-0.012	0.011	0.018	0.027	0.042	-0.038	-0.013	0.023	-0.015	-0.002	0.038	-0.013	-0.011	-0.048	-0.007	-0.032	-0.036	-0.006	-0.020
ERI3	-0.006	-0.031	-0.036	-0.026	-0.038	-0.004	-0.002	-0.035	-0.055	-0.040	-0.065	-0.039	0.029	-0.020	0.011	-0.062	0.020	-7.85e−4	-0.026	-0.006	0.007
ERI4	-0.004	0.044	0.029	0.017	0.015	0.002	-0.066	0.037	0.041	-0.040	0.067	0.053	0.026	0.009	0.050	0.037	-0.009	0.008	0.018	0.052	0.009
ERI5	-0.019	-0.011	-0.010	-0.021	-0.015	-0.053	-0.112	-0.015	0.039	-0.082	0.048	-6.40e−4	0.004	0.025	0.029	-0.007	0.013	0.056	0.022	0.079	0.023
ERI6	0.037	-0.003	0.044	0.014	-0.002	0.009	-0.024	0.053	0.068	-0.035	0.058	0.009	0.000	0.018	-0.012	0.038	-0.031	0.003	-0.046	0.057	-0.029
ERI7	-0.017	-0.016	-0.029	-0.022	-0.010	-0.039	-0.077	-0.003	0.036	-0.051	0.041	-0.018	-0.022	-2.79e−4	0.003	0.022	-0.008	0.057	-0.005	0.043	0.014
ERI8	-0.004	0.011	0.001	0.021	0.012	-5.38e−4	-0.078	0.052	0.070	-0.026	0.036	0.017	-0.045	-0.006	0.005	-0.001	-0.052	0.003	-0.047	0.037	-0.032
ERI9	-0.014	0.022	-0.014	0.028	-0.006	0.000	-0.099	0.017	0.060	-0.038	0.025	0.023	-0.035	0.001	0.040	-0.017	-0.057	-0.001	-0.043	0.040	-0.050
ERI10	-0.016	0.014	0.000	0.022	-0.006	-0.005	-0.091	0.016	0.032	-0.061	0.029	0.008	-0.051	-0.020	0.009	-0.015	-0.037	0.004	-0.048	0.036	-0.033
WFCt1	0	-0.128	0.078	-0.190	-0.022	-0.176	0.012	0.020	-0.011	-0.023	-0.030	-0.087	0.077	0.085	-0.048	-0.033	0.031	-0.009	-0.004	-0.031	0.015
WFCt2	-0.128	0	-0.123	0.094	0.032	0.011	-0.009	-0.083	0.008	0.025	-0.002	-0.015	0.014	0.053	-0.032	0.012	0.061	0.023	0.019	-0.019	0.061
WFCs1	0.078	-0.123	0	-0.179	0.014	-0.188	0.043	0.021	0.006	0.012	0.020	-0.058	0.073	0.076	-0.036	0.016	0.049	0.030	0.024	-0.015	0.051
WFCs2	-0.190	0.094	-0.179	0	-0.022	0.077	0.001	-0.106	-0.019	0.046	-0.014	-0.012	-0.032	0.015	-0.070	0.028	-0.009	-0.032	-0.026	-0.061	-0.017
WFCb1	-0.022	0.032	0.014	-0.022	0	-0.037	0.053	-0.034	0.002	0.053	0.001	-0.046	0.015	0.008	-0.032	-0.014	0.025	-0.001	0.014	-0.044	0.045
WFCb2	-0.176	0.011	-0.188	0.077	-0.037	0	0.092	-0.028	0.009	0.093	0.049	0.022	-0.028	-0.018	-0.102	-0.004	-0.011	-0.048	-0.014	-0.085	-0.017
BO1	0.012	-0.009	0.043	0.001	0.053	0.092	0	-0.185	-0.232	0.131	-0.158	-0.253	-0.034	-0.045	-0.146	-0.047	-0.030	-0.039	-0.012	-0.120	-0.017
BO2	0.020	-0.083	0.021	-0.106	-0.034	-0.028	-0.185	0	0.106	-0.254	0.077	0.001	0.075	0.109	-0.051	-0.031	0.051	-0.008	0.015	-0.055	0.010
BO3	-0.011	0.008	0.006	-0.019	0.002	0.009	-0.232	0.106	0	-0.290	0.039	0.082	0.008	0.040	-0.013	-0.007	0.093	0.068	0.055	-0.038	0.056
CF1	-0.023	0.025	0.012	0.046	0.053	0.093	0.131	-0.254	-0.290	0	-0.107	-0.140	-0.073	-0.073	-0.141	0.013	-0.038	-0.063	-0.070	-0.115	-0.035
CF2	-0.030	-0.002	0.020	-0.014	0.001	0.049	-0.158	0.077	0.039	-0.107	0	0.128	0.124	0.123	0.085	-0.008	0.057	0.030	0.055	0.005	0.074
CF3	-0.087	-0.015	-0.058	-0.012	-0.046	0.022	-0.253	0.001	0.082	-0.140	0.128	0	-0.017	-0.008	-0.012	0.044	0.031	0.021	0.031	-0.031	0.044
CS1	0.077	0.014	0.073	-0.032	0.015	-0.028	-0.034	0.075	0.008	-0.073	0.124	-0.017	0	0.062	-0.050	-0.118	-0.010	-0.019	-0.030	0.001	-0.011
CS2	0.085	0.053	0.076	0.015	0.008	-0.018	-0.045	0.109	0.040	-0.073	0.123	-0.008	0.062	0	-0.093	-0.074	0.020	0.010	-0.024	0.039	0.020
CS3	-0.048	-0.032	-0.036	-0.070	-0.032	-0.102	-0.146	-0.051	-0.013	-0.141	0.085	-0.012	-0.050	-0.093	0	0.035	0.020	0.027	-0.012	0.054	0.024
SS1	-0.033	0.012	0.016	0.028	-0.014	-0.004	-0.047	-0.031	-0.007	0.013	-0.008	0.044	-0.118	-0.074	0.035	0	0.029	-8.07e−4	-0.018	0.048	-0.032
SS2	0.031	0.061	0.049	-0.009	0.025	-0.011	-0.030	0.051	0.093	-0.038	0.057	0.031	-0.010	0.020	0.020	0.029	0	0.019	-0.002	-0.054	0.012
SS3	-0.009	0.023	0.030	-0.032	-0.001	-0.048	-0.039	-0.008	0.068	-0.063	0.030	0.021	-0.019	0.010	0.027	-8.07e−4	0.019	0	0.022	-0.052	-0.021
SS4	-0.004	0.019	0.024	-0.026	0.014	-0.014	-0.012	0.015	0.055	-0.070	0.055	0.031	-0.030	-0.024	-0.012	-0.018	-0.002	0.022	0	-0.018	-0.006
SS5	-0.031	-0.019	-0.015	-0.061	-0.044	-0.085	-0.120	-0.055	-0.038	-0.115	0.005	-0.031	0.001	0.039	0.054	0.048	-0.054	-0.052	-0.018	0	0.027
SS6	0.015	0.061	0.051	-0.017	0.045	-0.017	-0.017	0.010	0.056	-0.035	0.074	0.044	-0.011	0.020	0.024	-0.032	0.012	-0.021	-0.006	0.027	0
